# A Design Pattern for Decentralised Decision Making

**DOI:** 10.1371/journal.pone.0140950

**Published:** 2015-10-23

**Authors:** Andreagiovanni Reina, Gabriele Valentini, Cristian Fernández-Oto, Marco Dorigo, Vito Trianni

**Affiliations:** 1 IRIDIA, Université Libre de Bruxelles, Brussels, Belgium; 2 Faculté des Sciences, Université Libre de Bruxelles, Brussels, Belgium; 3 ISTC, Consiglio Nazionale delle Ricerche, Rome, Italy; Kyushu University, JAPAN

## Abstract

The engineering of large-scale decentralised systems requires sound methodologies to guarantee the attainment of the desired macroscopic system-level behaviour given the microscopic individual-level implementation. While a general-purpose methodology is currently out of reach, specific solutions can be given to broad classes of problems by means of well-conceived *design patterns*. We propose a design pattern for collective decision making grounded on experimental/theoretical studies of the nest-site selection behaviour observed in honeybee swarms (*Apis mellifera*). The way in which honeybee swarms arrive at consensus is fairly well-understood at the macroscopic level. We provide formal guidelines for the microscopic implementation of collective decisions to quantitatively match the macroscopic predictions. We discuss implementation strategies based on both homogeneous and heterogeneous multiagent systems, and we provide means to deal with spatial and topological factors that have a bearing on the micro-macro link. Finally, we exploit the design pattern in two case studies that showcase the viability of the approach. Besides engineering, such a design pattern can prove useful for a deeper understanding of decision making in natural systems thanks to the inclusion of individual heterogeneities and spatial factors, which are often disregarded in theoretical modelling.

## Introduction

Large-scale decentralised systems are becoming the more ubiquitous the more our lives become connected, and the problem of understanding and controlling such complex systems is currently approached from a variety of perspectives [1–3]. A particularly interesting viewpoint on decentralised systems is the one of Cognitive Science, which looks at their ability of collecting, transforming and propagating information within the system while interacting with the external world [4, 5]. However, providing desired cognitive processing abilities to large-scale distributed systems—from swarms of robots [6] to cognitive radio networks [7] and cyber-physical systems [8]—is a complex problem that has no general solution available to date. This is mainly due to difficulties in treating at the same time several complexity factors (e.g., heterogeneities in the interaction topology or in the individual behaviour). Additionally, each application faces domain-specific challenges. For instance, in swarm robotics, any design methodology needs to deal with the inherent spatial factors [9–12].

In lieu of a universal design methodology, general solutions can be provided for specific classes of problems exploiting the concept of design patterns well known in software engineering [13]. Design patterns provide formal guidelines to deal with recurring problems in a specific field. For distributed systems, design patterns prescribe the individual-level microscopic behaviour required to obtain desired system-level macroscopic properties [14–16]. According to the literature, design patterns are defined by the following attributes: name, context, design rationale, problem and solution (see S1 Text). Each attribute identifies and formally describes one component of the proposed design method. For instance, the context determines the domain of applicability of the design pattern, while the design rationale explains the what and the how, that is, the origins and working principles. Additionally, one or more case studies illustrate the application of the proposed solution to a specific problem instance.

Among the classes of cognitive processes that can be addressed through well-conceived design patterns, decentralised decision making represents a fundamental ability in several contexts and application domains [17–20]. In this paper, we propose a design pattern for decentralised decision making based on the nest-site selection behaviour of honeybee swarms [21–23]. Previous experimental and theoretical studies have demonstrated near-optimal speed-accuracy tradeoffs in the selection of the most profitable option among a set of alternative nesting sites by honeybees [21, 22]. Most importantly, inhibitory signals among bees provide an adaptive mechanism to quickly break deadlocks and tune the decision dynamics according to the perceived quality of the discovered options [22, 23]. The above properties of the nest-site selection process are relevant for many practical decision-making scenarios in decentralised systems, and justify its choice in this study.

Starting from the macroscopic description of the nest-site selection dynamics [22, 23], we derive the exact relationship between microscopic and macroscopic models—also including finite-size effects—for the general case of a best-of-*n* decision problem. The inter-related models represent the core of the design pattern, which is completed by formal guidelines for the implementation of collective decisions in multiagent systems. We provide guidelines for implementation by means of either homogenous or heterogeneous agents, as well as guidelines for the inclusion of spatial and topological factors that have a bearing in determining the microscopic interaction patterns [9]. We illustrate the application of the design pattern in two case studies, covering decentralised decisions by (i) static agents interacting in a fully-connected network, and (ii) mobile agents involved in a search and exploitation task.

## Collective Decisions through Cross Inhibition

We consider a best-of-*n* decision problem, that is, the choice of the best option, or any of the equal-best options, among *n* different alternatives. Each option *i* ∈ {1, …, *n*} is characterised by a quality *v*_*i*_ ∈ [*v*_*m*_, *v*_*M*_]. We study decision making for a population A of *N* agents, where each agent *a*_*g*_, *g* ∈ {1, …, *N*} is either committed to one of the available options *i* and belongs to the sub-population $A_{i}$ (size *N*_*i*_ and fraction Ψ_*i*_ = *N*_*i*_/*N*) or is uncommitted and belongs to sub-population U (size *N*_*U*_ and fraction Ψ_*U*_). Agents can obtain a noisy estimate ${\hat{v}}_{i}$ of the quality associated to option *i*. At the macroscopic level, a decision is taken as soon as the entire population or a large fraction Ψ_*q*_ (hereafter referred to as *quorum*) is committed to a single option.

In this section, we first present a macroscopic model that accounts for the system mean-field dynamics, and that describes the decision process at the population level without considering the actions of each individual agent and their inherent stochasticity. Then, we introduce a microscopic model in the form of a probabilistic finite state machine (PFSM) to describe the behaviour of the individual agent that changes its commitment state in response to probabilistic events. This description level is the closest to the agent implementation and allows us to directly link the control parameters of the agent behaviour to the macroscopic parameters. Finally, we provide the link between macro and micro levels through equations that convert ODE transition rates into the PFSM probabilities.

### Macroscopic model

According to the model proposed for honeybee nest-site selection [22], four concurrent processes determine the distribution of agents across populations: (i) uncommitted agents spontaneously *discover* option *i* at rate *γ*_*i*_ (ii) agents committed to option *i* spontaneously *abandon* commitment at rate *α*_*i*_; (iii) agents committed to option *i* *recruit* uncommitted agents at rate *ρ*_*i*_; and (iv) agents committed to option *j* ≠ *i* *inhibit* agents committed to option *i* at rate *σ*_*j*_ (cross-inhibition). A committed agent that receives an inhibitory signal stops recruiting (hence the expression *stop signal*) and reverts to the uncommitted state, becoming available for discovery or recruitment by other agents. Such cross-inhibition mechanism allows the swarm to tune the decision speed and break decision deadlocks in case of equal-best options [22].

The mean-field macroscopic dynamics are well described by an *n*-dimensional ODE system, which extends the previously studied binary version [22, 23]:

{Ψ˙i=γiΨU-αiΨi+ρiΨiΨU-∑j≠iσjΨiΨjΨU=1-∑iΨi} (1)

where *i* ∈ {1, …, *n*}. Here, the variation of Ψ_*i*_ is determined by the four processes described above, and a mass conservation equation constrains the dynamics within feasible bounds. The transition rates (*γ*_*i*_, *α*_*i*_, *ρ*_*i*_ and *σ*_*i*_) are functions of the quality *v*_*i*_:

$$\alpha_{i}=f_{\alpha }\left(v_{i}\right),\;\gamma_{i}=f_{\gamma }\left(v_{i}\right),\;\rho_{i}=f_{\rho }\left(v_{i}\right),\;\sigma_{i}=f_{\sigma }\left(v_{i}\right)$$ (2)

The relations between option quality and transition rates determine the macroscopic dynamics [23].

### Microscopic model

**Fig 1:**
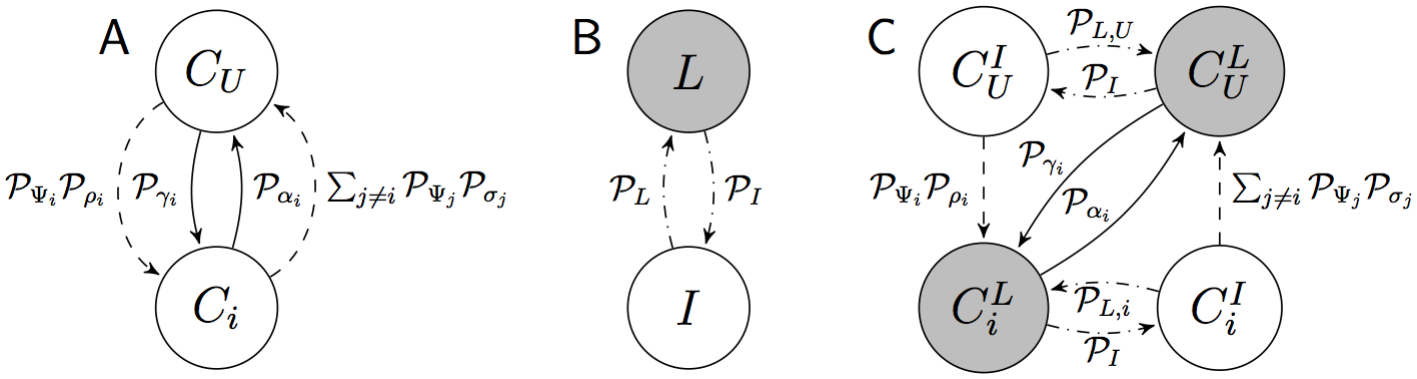
Probabilistic Finite State Machines (PFSMs) describing the microscopic behaviour of an agent in average. Here, the notation $P_{\lambda_{i}}$ is a shorthand for $P_{\lambda }\left(v_{i}\right)$ (A) PFSM describing the basic commitment dynamics. Spontaneous transitions are represented by solid lines, interactive transitions by dashed lines. For a complete version with *n* + 1 states, see panel A in S1 Fig (B) PFSM describing the dynamics of activity change as agents switch from latent to interactive states. Latent states are indicated in grey, while changes in the activity state are represented by dash-dotted arrows. (C) PFSM describing the coupled dynamics of commitment and activity change. For a complete version with 2(*n* + 1) states, see panel B in S1 Fig.

The behaviour of individual agents is represented by the probabilistic finite state machine (PFSM) shown in Fig 1A that describes the *commitment dynamics*. An agent can be either uncommitted (state *C*_*U*_) or committed to option *i* (state *C*_*i*_), and probabilistically changes state every *τ* seconds according to two types of transitions: spontaneous and interactive. Spontaneous transitions model the discovery of option *i* with probability $P_{\gamma }\left(v_{i}\right)$ and the abandonment of commitment to option *i* with probability $P_{\alpha }\left(v_{i}\right)$. Interactive transitions model the recruitment and cross-inhibition processes resulting from the interaction between agents belonging to different populations. We refer to the probability of any agent interacting with an agent committed to option *i* as $P_{\Psi_{i}}$. We assume a well-mixed system, so that $P_{\Psi_{i}}=N_{i}/N$. Recruitment for option *i* is modelled by a transition from *C*_*U*_ to *C*_*i*_ with overall probability $P_{\Psi_{i}}P_{\rho }\left(v_{i}\right)$. Cross-inhibition of an agent committed to option *i* is instead modelled as the cumulative effect of the interaction with agents committed to a different option, with overall probability $\sum_{j\neq i}P_{\Psi_{j}}P_{\sigma }\left(v_{j}\right)$ (see S1 Text for details).

### Micro-macro link

The PFSM of Fig 1A represents the average agent behaviour (see also S1 Fig) [24]. Its actual implementation requires choosing the way in which transitions are executed in relation to the limited information available to the individual agent. For instance, the estimation of the population-size dependent probability $P_{\Psi_{i}}$ by individual agents requires some sampling of the current population size. The implementation is rather straightforward, and is detailed in S1 Text. Here, we focus on the other transition probabilities ($P_{\gamma }$, $P_{\alpha }$, $P_{\rho }$, $P_{\sigma }$), and their correspondence with the macroscopic transition rates to obtain the desired dynamics determined by Eq (2).

We propose two strategies based either on a *homogeneous* or on a *heterogeneous* implementation. In the homogeneous case, all agents compute their transition probabilities in the same way as a function of the estimated quality ${\hat{v}}_{i}$. In this case, it is possible to establish a direct correspondence between micro and macro parameters:

$$P_{\lambda ,g}\left({\hat{v}}_{i}\right)=\lambda_{i}\tau =f_{\lambda }\left({\hat{v}}_{i}\right)\tau ,\;\begin{array}{c} i\in \{1,\cdots ,n\}\\ \lambda \in \{\gamma ,\alpha ,\rho ,\sigma \} \end{array}$$ (3)

where *P*_*λ*,*g*_ represents the actual probability for the agent *a*_*g*_ to undergo the transition *λ*. Hence, a principled choice of the individual transition probabilities can be made to obtain the desired macroscopic dynamics as defined by Eq (2). The derivation of the micro-macro link is detailed in S1 Text.

In the heterogeneous case, each agent computes its own transition probabilities differently from other agents. We propose a simple response threshold scheme [25], so that agent *a*_*g*_ follows a transition with a fixed probability if the (estimated) option quality ${\hat{v}}_{i}$ exceeds a given threshold *δ*_*g*_:

$$P_{\lambda ,g}\left({\hat{v}}_{i}\right)=\{\begin{array}{cc} P_{\lambda ↑} & \hat{v_{i}}>\delta_{g}\\ P_{\lambda ↓} & \hat{v_{i}}\leq \delta_{g} \end{array},\;\begin{array}{c} i\in \{1,\cdots ,n\}\\ \lambda \in \{\gamma ,\alpha ,\rho ,\sigma \} \end{array}$$ (4)

where $P_{\lambda ↑}$ and $P_{\lambda ↓}$ are tuneable parameters, and the value *δ*_*g*_ is drawn for each agent *a*_*g*_ from a probability distribution $D_{\lambda }$ over the range [*v*_*m*_, *v*_*M*_]. With this implementation, it is possible to establish a relationship between microscopic and macroscopic parameters through the cumulative distribution function $F_{D_{\lambda }}$ of $D_{\lambda }$:

$$F_{D_{\lambda }}=\frac{\lambda \tau -P_{\lambda ↓}}{P_{\lambda ↑}-P_{\lambda ↓}},\;\lambda \in \left\{\gamma ,\alpha ,\rho ,\sigma \right\}$$ (5)

For $F_{D_{\lambda }}$ to be a cumulative distribution function, the relationship between quality and macroscopic transition rate expressed in Eq (2) must be monotonic in *v*—either increasing or decreasing. As a consequence, the step function Eq (4) can be determined by:

$$\begin{array}{c} P_{\lambda ↑}=f_{\lambda }\left(v_{M}\right)\tau \\ P_{\lambda ↓}=f_{\lambda }\left(v_{m}\right)\tau \end{array}\;\lambda \in \left\{\gamma ,\alpha ,\rho ,\sigma \right\}$$ (6)

The micro-macro correspondence given by Eqs (5) and (6) holds when each agent *a*_*g*_ re-samples the threshold *δ*_*g*_ from $D_{\lambda }$ at every decision step (see also S1 Text). An approximation with fixed thresholds is valid for recruitment and cross-inhibition, because re-sampling is ensured by changing partner in each different interaction (as shown in case study I-A below). Fixed thresholds for spontaneous transitions should not be used, unless the macroscopic dynamics are dominated by recruitment and cross-inhibition (see case study I-B).

For both strategies—homogeneous and heterogeneous—the derivation of the relationship between microscopic and macroscopic description levels passes through the introduction of a finite-size stochastic macroscopic model determined by the system Master equation, which allows us to link the microscopic stochastic description with the macroscopic mean-field dynamics (see S1 Text for a detailed description).

### Latent and interactive agents

In a practical application scenario, agents might not be able to interact with neighbours every *τ* seconds. For instance, an agent might be busy estimating the quality of a discovered option, or spatial/topological factors might prevent frequent interactions. Agents unable to interact are latent, as opposed to interactive ones. We model changes in this activity state (i.e., the *activity dynamics*) by considering that an agent becomes latent with probability $P_{L}$, and returns interactive with probability $P_{I}$. The switch in activity state is represented by the PFSM in Fig 1B, which predicts that a fraction of $\eta_{I}=P_{I}/\left(P_{I}+P_{L}\right)$ agents can be found asymptotically in the interactive state. Similarly, $\eta_{L}=P_{L}/\left(P_{I}+P_{L}\right)$ represents the fraction of agents in the latent state. The activity switch is possible for both committed and uncommitted agents, leading to a PFSM description with 2(*n* + 1) states (see S1 Text). Recruitment and cross-inhibition are available only to interactive agents, while discovery and abandonment are available also to latent agents, depending on the particular application. As an example, Fig 1C reports a PFSM that correctly preserves the micro-macro link, and is exploited for the search and exploration task discussed below. Given the microscopic description, it is possible to derive the correspondence between micro and macro parameters by dividing the macroscopic transition rates by *η*_*I*_ or *η*_*L*_ in Eqs (3), (5) and (6). For the specific case shown in Fig 1C, recruitment and cross-inhibition are modulated by *η*_*I*_, discovery and abandonment by *η*_*L*_ (see S1 Text).

## Results

Following the implementation guidelines described above, we present here two case studies that showcase the usage of the proposed design pattern. Case study I concerns decentralised decisions-making by static agents interacting on a fully-connected network, and is divided in two parts, I-A and I-B. Case study II concerns collective decisions by mobile agents in the context of a search and exploitation task. To ease the discussion and simplify the visualisation, we present here a binary decision problem (options A and B with quality *v*_*A*_ and *v*_*B*_), and we provide additional results for the best-of-*n* scenario in the online supplementary material. To quantify the agreement between macroscopic models and microscopic implementation, we look at the system performance through a set of metrics detailed below, and we compare the process dynamics at different abstraction levels.

### Metrics

Different metrics are used in the literature to evaluate the results of a decision-making process, which are linked to the correctness of the response, the coherence across multiple repetitions, as well as the speed of the process. Whenever time is required to gather sufficient information, decision making gives rise to speed-accuracy tradeoffs—a very common phenomenon in biological systems [26]. In collective decision making, a high coherence of the group is also important, as it can minimise the costs for conflicting choices by individuals. As maintaining coherence is a time-consuming process that requires to spread information widely within the group, speed-cohesion tradeoffs may also appear [27].

In decentralised systems, accuracy can be defined as the proportion of the group that is committed to the best option, or to any of the equal-best options. Conversely, cohesion measures the ability of the group to be committed to the same option, notwithstanding its quality [27]. Therefore, one can simultaneously have low accuracy and high cohesion, for instance if 10% of the group choses a high-quality option and the remaining 90% goes for a low-quality one. In the context of engineering artificial systems, it is important to quantify both the aspects of decision accuracy and group cohesion, and constrast them with the time required to arrive at a decision.

To this purpose, we first introduce the **resolution** R, which refers to the ability to discriminate between different-quality options, and is related to the normalised quality difference between any two options *A* and *B*: $R=|v_{A}-v_{B}|/\max\left(v_{A},v_{B}\right)$. By requiring a target resolution, the designer can focus on maximising accuracy only in those portions of the problem space that are above resolution, and just require that any solution is chosen below the given resolution threshold. Note that resolution is normalised so that the minimum quality difference that can be detected is proportional to the quality magnitude, in analogy to many biological decision-making processes following the Weber’s law.

Then, we consider the **effectivity** E as the ability of the group to take a decision within the maximum execution time *T*. The effectivity is measured as the fraction of runs that reach the quorum Ψ_*q*_ within the given time limit. Effectivity is related to the coherence of decision making, as it measures the ability to take a decision (i.e., reach the predefined quorum) within the maximum allotted time, notwithstanding the quality of the chosen option. By requiring a minimum effectivity threshold, the designer can impose that the system reaches a coherent state within a maximum time *T*.

Having defined resolution and effectivity, we introduce the main performance metrics we take into account. The **success rate** S corresponds to the fraction of effective runs resulting in a correct decision—i.e., the quorum is reached for the best option, or any of the equal-best options—when starting from a fully-uncommitted population. The success rate is defined in analogy to the exit probability in stochastic processes, and is related to the accuracy of decision making, because the quality of the chosen option is taken into account. Note that, by looking at the effective runs only—e.g., runs with effectivity larger than the given threshold—we limit ourselves only to high-coherence results. The **convergence time** C is the average time required to reach the quorum Ψ_*q*_ computed over all effective runs, and is defined in analogy to the exit time of stochastic processes. This metric actually corresponds to the speed of the decision-making process, and can be exploited together with the success rate to select the most convenient solution that optimises the speed-accuracy/cohesion tradeoff.

### Case study I: Collective decisions on a fully-connected network

The first case study illustrates the implementation of decentralised decision making for a multiagent system in which each agent can potentially interact with any other agent (see Methods). We present two parameterisations as case study I-A and I-B.

In Case study I-A, we study consensus decisions, that is, we design a system in which the desired outcome is complete convergence of the group towards the choice of one or the other option. To this end, we set the decision quorum Ψ_*q*_ = 1 and we require that a decision is taken within *T* = 400*s*. Here, we also assume that option quality varies in *v* ∈ [0, 1].

The first step towards implementation is the definition of the macroscopic parameterisation and its relationship with the option quality. The analysis of the macroscopic dynamics from Eq (1) reveals that consensus can be achieved only when abandonment is null (*α*_*A*_ = *α*_*B*_ = 0). We arbitrarily choose a constant cross-inhibition rate $\sigma_{A}=\sigma_{B}=\bar{\sigma }$, which is sufficient for determining a collective decision [23]. The value $\bar{\sigma }$ can be tuned to determine the time scale of the process: the higher the rate, the quicker the convergence dynamics. Here, we choose $\bar{\sigma }=1$. In these conditions, the model predicts two equilibrium points corresponding to consensus decision for either of the two options, but their stability may vary depending on the relative strength of discovery and recruitment. Assuming *v*_*A*_ ≥ *v*_*B*_, the Model (1) predicts that the equilibrium at consensus for A is always stable, while consensus for B is stable only when *γ*_*A*_ < *γ*_*B*_ + *ρ*_*B*_ (see S2 Text). Thanks to this result, an informed choice can be made about the macroscopic parameterisation and the relation with option quality: *γ*_*i*_ = *f*_*γ*_(*v*_*i*_), *ρ*_*i*_ = *f*_*ρ*_(*v*_*i*_), *i* ∈ {A, B}. In particular, assuming a target resolution *R* = 0.15, we can minimise the chances of a wrong decision by designing the system to have a single stable equilibrium for the best option in any decision problem characterised by above-resolution quality differences. We select linear functions that link macroscopic transition rates to the quality:

$$f_{\gamma }\left(v_{i}\right)=k\;v_{i},\;f_{\rho }\left(v_{i}\right)=h\;v_{i}$$ (7)

where *k* and *h* are tuneable parameters. Next, we compute the constraint on the above functions to satisfy our design choice: *k* > *h* (1 − *R*)/*R* (see S2 Text). Finally, we choose values that comply with the prescribed bounds: *h* = 0.1 and *k* = 0.6.

The second step towards implementation is the analysis of the system performance in the complete decision space for varying system size *N*. This can be studied numerically by approximating the finite-size macroscopic dynamics using the Gillespie algorithm [28]. Finally, the multiagent system can be deployed following the prescriptions of the design pattern and choosing a convenient implementation strategy. In the homogeneous case, all agents determine *P*_*γ*_ and *P*_*ρ*_ in the same way according to Eqs (3) and (7). Conversely, in the heterogeneous case transition probabilities are determined by the step function of Eq (4), and vary from agent to agent with thresholds randomly sampled from the distribution determined by Eqs (5), (6) and (7). In this case study, we use fixed thresholds and we therefore limit the heterogeneous implementation to the recruitment probability *P*_*ρ*_, while we keep the discovery probability *P*_*γ*_ homogeneous across agents. Finally, we let agents update their state every *τ* = 0.2 *s* following the design pattern guidelines (see S1 Text).

**Fig 2:**
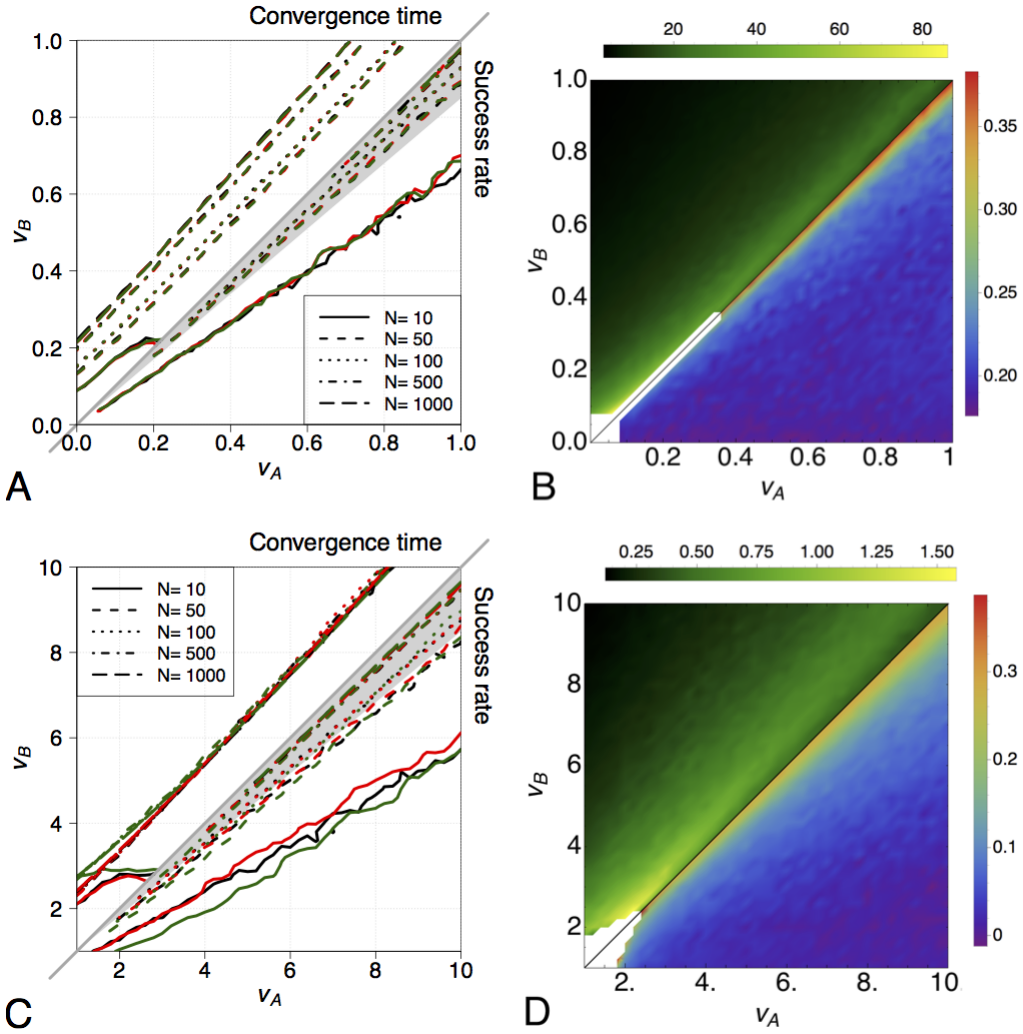
Collective decisions on a fully-connected network: comparison between the micro and the macro dynamics and scaling properties. (*A*,*C*) Comparison between the stochastic finite-size macroscopic model (black lines) and the multiagent implementation with both the homogeneous strategy (red lines) and the heterogenous strategy (green lines). Results are displayed for varying system size *N*. For each possible configuration (*v*_*A*_, *v*_*B*_), 500 independent runs are performed. We show results for configurations with effectivity E>0.7. The plot is divided in two parts: in the bottom-right triangle, we consider the success rate S for each configuration (*v*_*A*_, *v*_*B*_), where *v*_*A*_ ≥ *v*_*B*_. For each group size *N*, we show the isolines at S=0.9. The gray triangle indicates quality value pairs below the target resolution *R* = 0.15 (i.e., configurations in which the two options are considered equivalent). In the top-left half of the plot, we consider the convergence time C for each configuration (*v*_*A*_, *v*_*B*_), where *v*_*B*_ ≥ *v*_*A*_ (i.e., the symmetric problems with respect to the bottom-right plot). For each group size *N*, we show the isolines at $C=\hat{C}$. (*B*, *D*) Scaling of the convergence time C with the system size *N*. For each configuration (*v*_*A*_, *v*_*B*_), we fit the curve $C=b\;N^{a}$ and we show the heat-map for the fitted coefficient *a* (see the bottom-right triangle showing the coefficient value for each configuration (*v*_*A*_, *v*_*B*_), *v*_*A*_ ≥ *v*_*B*_) and *b* (see the top-left triangle showing the coefficient value for symmetric configurations (*v*_*A*_, *v*_*B*_), *v*_*B*_ ≥ *v*_*A*_) across the decision space. Also in this case we show only configurations where E>0.7 for all *N*, and the white space indicates configurations with low effectivity. (*A*,*B*) Results for case-study 1A with *v*_*i*_ ∈ [0, 1], *γ*_*i*_ = 0.6*v*_*i*_, *α*_*i*_ = 0, *ρ*_*i*_ = 0.1*v*_*i*_, *σ*_*i*_ = 1 and *i* ∈ {A, B}, $\hat{C}=50s$. (*C*,*D*) Results for case-study 1B with *v*_*i*_ ∈ [1, 10], *γ*_*i*_ = *ρ*_*i*_ = *v*_*i*_, *α*_*i*_ = 1/*v*_*i*_, *σ*_*i*_ = 10 and *i* ∈ {A, B}, $\hat{C}=1s$.

The performance of the multiagent system for both homogenous and heterogeneous implementations is compared to the macroscopic Gillespie simulations for varying system size *N* (see Fig 2A). An excellent match between microscopic and macroscopic dynamics can be observed for every system size, for both the success rate S and the convergence time C. When the difference in quality between the two options is above the resolution *R* = 0.15, the correct decision is taken in at least 90% of the cases (i.e., S=0.9, as evidenced by the isolines in the bottom-right part of Fig 2A laying within the grey shaded area) for every system size but *N* = 10. Indeed, small groups suffer from stochastic fluctuations, reflected by a substantially lower success rate with respect to larger groups. Conversely, the speed of the process is lower for larger groups, as indicated by the isolines for $\hat{C}=50s$ in the top-left part of Fig 2A. To quantify the scaling properties with respect to the system size, we analysed the convergence time for each decision problem as a function of *N*. We found a generalised adherence with a power law behaviour $C=b\;N^{a}$, with exponent *a* ≈ 0.2 as shown in Fig 2B for macroscopic Gillespie simulations, and S2 Fig for multiagent simulations. The coefficient *b* also varies with the decision problem: the lower the option quality difference, the higher the coefficient. Looking at Fig 2B and S2 Fig, we observe that C scales similarly across different decision problems, with the exception of problems characterised by similar qualities (i.e., *v*_*A*_ ≈ *v*_*B*_) that require in general more time for convergence. Finally, we show in S3 Fig an example of the convergence dynamics for a specific decision problem (e.g., *v*_*A*_ = 0.9, *v*_*B*_ = 0.6), which highlights the close correspondence between ODEs, Gillespie and multiagent simulations.

Case study I-B is concerned with the general case of value-sensitive decision making [23], and discusses the implementation in case of less restricting conditions with respect to the previous case study. We consider a quality range *v* ∈ [1, 10], we fix the quorum for the collective decision to Ψ_*q*_ = 0.8 and we limit the total execution time to *T* = 40 s. Here, we also demonstrate a fully heterogeneous implementation of the multiagent system.

To obtain value-sensitive decision making [23], discovery and recruitment rates are assumed to be linearly proportional to the option quality *v*_*i*_ (i.e., *γ*_*i*_ = *ρ*_*i*_ = *v*_*i*_, *i* ∈ {A, B}), the abandonment rate is inversely proportional to quality (i.e., *α*_*i*_ = 1/*v*_*i*_), while the cross-inhibition rate is constant ($\sigma_{i}=\bar{\sigma }$), which we fix to $\bar{\sigma }=10$. Given the macroscopic parameterisation, we follow the same methodology described above to analyse the finite-size effects produced by the system size *N*, and we design the multiagent system following both the homogeneous and the heterogeneous strategies (see Methods). In the latter case, we use fixed thresholds for all transition probabilities and discuss the error introduced with respect to the macroscopic dynamics. According to the design pattern prescriptions, agents are updated every *τ* = 20 ms (see S1 Text).

Fig 2C shows the match between the macroscopic Gillespie simulations and the multiagent implementation with both the homogeneous and the heterogeneous strategy, for varying system size *N* (see also S3 Fig for an example of convergence dynamics). The correspondence between macroscopic model and microscopic implementation is remarkable also in this case. Even with the rough approximation of fixed thresholds for the heterogeneous case, we note a good match of the micro-macro dynamics, although not perfect especially for the convergence time C at low qualities. The results show that the studied parameterisation allows to reliably take decisions for above-resolution decision problems already with *N* = 100, as indicated by the success rate S in the bottom-right part of Fig 2C. Conversely, the convergence time C is very similar across different system sizes. Also in this case, we analysed the scaling behaviour of the convergence time, and found adherence with a power law behaviour $C=b\;N^{a}$, but with a very low exponent *a* (see Fig 2D for Gillespie simulations and S2 Fig for multiagent simulations). With the proposed parameterisation, C becomes nearly independent of the system size *N* in large parts of the problem space. The coefficient *b* is rather low too, indicating fast decisions especially for large differences in quality. This is a result of the higher transition rates chosen for the macroscopic model, which are reflected by a smaller timestep *τ* in the multiagent implementation as prescribed by the design pattern. Finally, we studied the micro-macro link in a best-of-*n* scenario. The results presented in S4 Fig reveal a very good correspondence between multiagent and Gillespie simulations, therefore validating the methodology beyond the binary decision problems presented above.

### Case study II: Collective decisions in a search and exploitation problem

Here, we present a case study that includes as main features spatiality and local interactions between agents. The experimental scenario is similar to the swarm robotics study of [16]. Point-size agents move in a 2D environment characterised by three areas—a *home* and two *target* areas—and must select the best quality target (see Methods). Uncommitted agents explore the environment to discover new options. Committed agents recruit and cross-inhibit other agents and periodically re-estimate the quality of the option they are committed to. Differently from [16], here quality is independent of distance. However, spatiality may influence the decision dynamics (e.g., the rate of discovery is higher for closer targets), and only an accurate design of the agent behaviour can lead to the systematic choice of the best available option.

We choose a macroscopic parameterisation similar to the one of case study I-B, but option quality *v*_*i*_ varies in [0.1, 1] and also the cross-inhibition rate varies linearly with quality (i.e., *σ*_*i*_ = *v*_*i*_). Besides depending on option quality, discovery is episodic (see S1 Text), being determined by the diffusive motion pattern of uncommitted agents that start searching from home. We model the macroscopic discovery rate to be proportional to the quality *v*_*i*_ and to decay with the target distance *d*_*i*_ as follows:

$$\gamma_{i}=\frac{v_{i}\;\mu e^{-\xi d_{i}}}{d_{i}}$$ (8)

where *ξ* and *μ* are parameters estimated from preliminary experiments (*μ* ≈ 0.12 and *ξ* ≈ 0.24), although geometrical approximations could be used as well.

Besides discovery, spatiality influences also the interaction patterns among agents, given that interactions are possible only with agents in the local neighbourhood (see Methods). To ensure a well-mixed system and comply with the design pattern requirements, we limit interactions within the home area and we force agents to periodically return home. As described above, the design pattern prescribes to have fixed probabilities to become interactive (e.g., return home with probability $P_{I}$) or latent (e.g., leave home with probability $P_{L}$). Therefore, we designed the individual behaviour after the microscopic description of Fig 1C following both the homogeneous and the heterogeneous strategy, and we set $P_{I}=0.001$ and $P_{L}=9\;P_{I}$ to ensure a fraction *η*_*I*_ = 0.1 of interactive agents on average.

**Fig 3:**
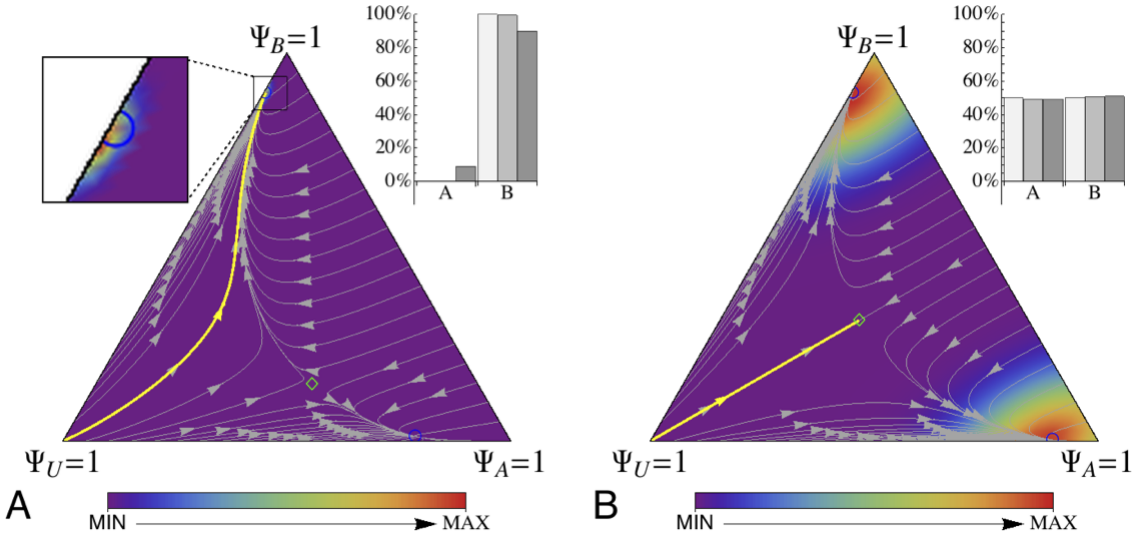
Collective decisions in a search and exploration problem: comparison between the micro and the macro dynamics. The state space of the system is presented as a ternary plot characterised by Ψ_*U*_ + Ψ_*A*_ + Ψ_*B*_ = 1, so that vertices correspond to fully-uncommitted or fully-committed populations. Macroscopic dynamics are indicated by trajectories and equilibrium points from the ODE model of Eq (1), parametrised according to the specific configuration. The bold yellow trajectory indicates the behaviour starting from a fully-uncommitted population (Ψ_*U*_ = 1). Stable equilibrium points are indicated as blue empty circles, while unstable points are indicated as green empty diamonds. The density map in the background represents the results of homogeneous multiagent simulations (1000 runs). The inset shows the success rate S for macroscopic Gillespie simulations (white bars) and multiagent simulations (homogeneous in light gray and heterogeneous in dark grey). (*A*) Micro-macro link for a decision problem in which the best option is also the farthest one (*v*_*A*_ = 0.7 < *v*_*B*_ = 1 and *d*_*A*_ = 1.5 m < *d*_*B*_ = 2.5 m). The magnify-glass effect allows to appreciate the close correspondence between the stable point predicted by the macroscopic model and the results from the multiagent simulations. (*B*) Micro-macro link for a completely symmetric decision problem (*v*_*A*_ = *v*_*B*_ = 1 and *d*_*A*_ = *d*_*B*_ = 2.5 m).

The design choices detailed above allow us to determine the microscopic parameterisation starting from the desired macroscopic transition rates (see S5 Fig for a comparison between the rates determined by design and those estimated from multiagent simulations through survival analysis [29]). We have tested the micro-macro link varying both option quality and target area distance, to observe how the implementation deals with the inclusion of spatial factors and interactive-latent dynamics. Indeed, the macroscopic model does not consider such factors, exception made for the model of discovery of Eq (8). In particular, we are interested in making consistent choices notwithstanding the target area distance. Fig 3A shows one such case for a homogeneous system in which the better option is also the farthest. The macroscopic model predicts convergence on the best-quality option (B in this case, see the trajectory starting from Ψ_*U*_ = 1), and the simulations are centred at the predicted stable point. Good agreement between macroscopic Gillespie simulations and multiagent implementation is observable also for the success rate S (see Fig 3A inset). For same quality options, the target area distance biases the choice towards the closer target area (see S6 Fig for the homogeneous case and S7 Fig for the heterogeneous case). When both distance and option quality are equal (i.e., a completely symmetric condition), the system converges toward the one or the other option with equal probability, as shown in Fig 3B for the homogeneous case. Here too, the adherence between microscopic and macroscopic dynamics is remarkable. All these tests have been performed with *N* = 500 agents. Good agreement is observed also for different system sizes, as shown in S8 Fig.

## Discussion

The design pattern methodology we propose provides a complete framework that allows to move from the choice of the macroscopic parameterisation down to the implementation of the individual behaviour. Each step is supported by the principled understanding of the causal relationship between microscopic choices and macroscopic effects. We have substantiated the methodology with case studies that, despite being idealised, contain all the ingredients to be taken as reference for practical applications. In this respect, the inclusion of latent states for individual agents is particularly important, as it allows to preserve the micro-macro link also when interactions are sporadic or when spatiality interferes with the well-mixed assumption [16]. Indeed, departures from the macroscopic predictions are expected in case of heterogeneous interaction topologies, as is the case for other ordering processes [30, 31]. In this case, the micro-macro link could be preserved through the inclusion of heterogeneous mean-field approximations, which can correct the departure from the assumed well-mixed condition at the macroscopic level [32]. Future work should aslo take into account the macroscopic effects of interactions over adaptive and multi-layer networks, in order to (i) take into consideration the variability of the topology of interactions with time [33] and (ii) allow for the existence of different layers of connectivity among agents, each pertaining to specific context-related properties [34].

Besides engineering, our results can be relevant for better understanding the behaviour of natural systems. With respect to honeybee nest-site selection, our results provide testable hypotheses about the implementation algorithm employed by individual bees in relation to the proposed macroscopic model [22, 23]. Our implementation algorithm is simpler than other individual-based approaches [35, 36], as it abstracts several details that require assumptions difficult to be verified experimentally. Similarly to previous studies, [36, 37], we have shown in the second case study that spatiality affects the outcome of the collective decision biasing it towards closer sites. In our implementation, this is mainly the result of the quicker discovery of closer sites, while different latencies (e.g., shorter travel times) play a negligible role, thanks to the fixed probability of becoming interactive required to preserve the micro-macro link (see S1 Text). Field experiments should be targeted to verify the existence of a tradeoff between distance and quality. Furthermore, the effects of finite-sized groups in the decision dynamics predicted by the stochastic macroscopic simulations adhere with studies about group-size effects in natural conditions [38]: the larger the swarm the more accurate its decision. The scaling of decision time with group size that we have highlighted here represents another interesting aspect to investigate with field experiments. Finally, the extent to which behavioural heterogeneities influence honeybee nest-site selection would be interesting to study, as genetic and molecular determinants of honeybee behaviour seem to play an important role [39, 40].

Behaviour heterogeneity in social systems is an important aspect not to be overlooked, as it can lead to interesting collective dynamics that are not attainable in fully homogeneous systems [41–43]. In our study, the choice of response thresholds for the heterogeneous implementation strategy is supported by the large literature on inter-individual variability in social insects [25, 44–47]. Recent studies have recognised the importance of including individual differences in behaviour—often referred to as personality or behavioural syndrome [48]—to better understand the collective dynamics [49, 50]. Here, we have highlighted the relationship between the distribution of individual thresholds and the collective response function, so that macroscopic predictions could be matched against estimates of the real threshold distribution [51]. We have also shown that fixed response thresholds well approximate the macroscopic dynamics especially for interactive processes like recruitment [25]. Associating fixed response thresholds with variable probability and intensity of responses may lead to more flexible and robust behaviour at the colony level [47]. Response thresholds well adhere with adaptive mechanisms for threshold adaptation [51, 52], allowing to finely tune the macroscopic response to match the statistical regularities that characterise the task. This adaptivity can result from evolutionary factors [53] as well as from development and learning [47]. Integrating adaptive mechanisms in the microscopic implementation could lead to improved performance [54, 55], and represents a natural extension for the proposed design pattern.

## Methods

### Multiagent simulations on fully-connected networks

We implemented a synchronous simulation for a multiagent system on a fully-connected network that directly derives from the design pattern implementation guidelines. At simulation start, each agent *a*_*g*_ estimates the quality ${\hat{v}}_{i}$ of all available options *i* ∈ {1, …, *n*}, and on that basis computes its own transition probabilities $P_{\lambda ,g}\left({\hat{v}}_{i}\right)$, with *λ* ∈ {*γ*, *α*, *ρ*, *σ*}. In the homogeneous case, these are computed in the same way for each agent according to the desired parameterisation, as prescribed by Eq (3). In the heterogeneous case, each agent *a*_*g*_ draws a random threshold *δ*_*g*_ from the random distribution *D*_*λ*_ and computes the transition probabilities as prescribed by Eq (4).

The simulation proceeds in discrete time steps of length *τ*. At each time step *t*, every agent updates its state following the PFSM of Fig 1A. All spontaneous transitions are always available. Conversely, interactive transitions depend on the interaction with a randomly selected partner, who shares its own commitment state and probabilities of recruitment and cross-inhibition. Given the well-mixed property ensured by the fully-connected topology, the population-dependent probabilities $P_{\Psi_{i}}$ are estimated by randomly choosing a different agent $a_{\hat{g}}$ as partner at each time step and checking its state: transitions are activated if the selected partner is committed to some option *i* (see S1 Text for details). In this case, the probability of recruitment $P_{\rho ,\hat{g}}$ and of cross-inhibition $P_{\sigma ,\hat{g}}$ are received from the selected partner, otherwise they are null. In this way, the agent *a*_*g*_ has complete information to update its commitment state.

### Particle-based multiagent simulations

Agents are point-size particles capable to move in a 2D environment. Movement is simulated through kinematic equations on the basis of the current agent speed *ν* and orientation *θ*. The environment is an infinite plane and does not contain any wall or obstacle. No collision or physical interference among agents is taken into account, and agents are free to move in any location of the 2D plane. The environment contains three circular areas with radius *r* = 0.3 m: home, target A and target B. Target areas are located at a variable distance *d*_*i*_ ∈ [1.5 m, 3.5 m], *i* ∈ {A, B}, from the home area. Each target area is further characterised by a quality *v*_*i*_ ∈ [0.1, 1]; each agent can individually estimate the target area quality when is inside the area.

Agents move at a constant speed *ν* = 0.01 m/s and communicate with their neighbours within an interaction range *d*_*I*_ = 0.6 m. Every *τ* = 1*s*, agents update both their state and their motion direction *θ* as detailed below.

#### Interactive and latent agents

As a consequence of local communications and of the distance between target areas, agents committed to different targets and uncommitted agents cannot always interact with each other. To ensure a well-mixed system, we limit interactions only when agents are within the home area. Agents remain in the home area for a time interval *τ*_*I*_ exponentially distributed with average $1/P_{L}$. When a timeout expires, agents get latent and leave the home area. Agents remain latent for a time interval *τ*_*L*_ exponentially distributed with average $1/P_{I}$. The motion pattern is conceived to ensure that agents are within the home area once they get interactive again (see below). To ensure that on average 10% of the agents are interactive within the home area, we set $P_{L}=9P_{I}$.

#### Motion pattern

The agent motion direction *θ* is determined by the current agent state. While moving, odometry sensors are exploited to track the position of known areas. In this case study, we model noiseless sensors. In a more realistic implementation, agent-to-agent communication can be exploited to compensate for odometry noise [56].

An uncommitted agent *a*_*g*_ (*C*(*a*_*g*_) = *C*_*U*_) explores the environment in search of target areas: when latent, it chooses a random direction and leaves home moving in a straight line. Upon finding target area *i*, the agent makes an estimate of the quality ${\hat{v}}_{i}$ and gets committed with probability $P_{\gamma ,g}\left({\hat{v}}_{i}\right)$ (which is computed according to the homogeneous or heterogeneous implementation strategy). Then, it stores the target area estimated quality and location—which is kept updated through odometry—and returns back home. If an uncommitted agent has explored the environment for [*τ*_*L*_/2]*s* without encountering any target area, it returns home to ensure that it gets interactive when already within the home area.

An agent *a*_*g*_ committed to option *i* (*C*(*a*_*g*_) = *C*_*i*_), while interactive, randomly moves within the home area to communicate with neighbours. When latent, it leaves home to return to the chosen target area *i* and re-estimate its quality ${\hat{v}}_{i}$. The agent returns home after *τ*_*L*_/2 s to become interactive within the home area. While latent, the committed agent abandons commitment with probability $P_{\alpha ,g}\left({\hat{v}}_{i}\right)$ and returns home. This probability is also computed according to either the homogeneous or the heterogeneous implementation strategy.

#### Interaction pattern

When interactive, all agents located in the home area can exchange short communication messages with a randomly chosen neighbour. These messages contain information on the agent state, which is used to estimate the population-dependent probabilities (see S1 Text for details).

When committed to option *i*, agent *a*_*g*_ also communicates the stored location of the target area and its own probability of recruitment $P_{\rho ,g}\left({\hat{v}}_{i}\right)$ and cross-inhibition $P_{\sigma ,g}\left({\hat{v}}_{i}\right)$, computed either with the homogeneous or the heterogeneous implementation strategy. Recruitment takes place if the partner is uncommitted, otherwise cross-inhibition takes place only if the distance between the target area locations internally stored by the interacting agents is larger than the target area radius *r*. In this way, cross-inhibition takes place only between agents committed to different options, as prescribed by the design pattern.

When the population is divided between interactive and latent agents, the design pattern prescribes that the dynamics of activity change must be faster than changes in the commitment state. To achieve this, we let agents interact only upon getting latent. By doing so, we guarantee that the fraction of interactive agents committed to option *i* is always an unbiased representation of the global fraction Ψ_*i*_ (see S1 Text).

## Supporting Information

S1 Text Detailed description of the proposed design pattern methodology. In this document, we detail and formalise all aspects of the design pattern for decentralised decision making, in some cases repeating and extending the information succinctly given in the main text.
(PDF)

S2 Text Stability analysis and parameterisation choice for multiagent simulations on fully-connected networks. In this document, we provide additional results supporting case study I-A, in particular concerning the stability analysis of the macroscopic system and the selection of the desired macroscopic parameterisation.
(PDF)

S1 FigProbabilistic Finite State Machines (PFSMs) describing the microscopic behaviour of an agent in average.Here, the notation $P_{\lambda_{i}},\lambda \in \left\{\gamma ,\alpha ,\rho ,\sigma \right\}$, *i* ∈ {1, …, *n*} is a shorthand for $P_{\lambda }\left(v_{i}\right)$. (A) PFSM describing the basic commitment dynamics for *n* possible options. Spontaneous transitions are represented by solid lines, while interactive transitions are represented by dashed lines. (B) PFSM describing the coupled commitment and activity dynamics. Latent states are indicated in grey, and dash-dotted lines represent changes between latent and interactive states.(TIFF)

S2 FigScaling of the convergence time C with the system size *N*.For each configuration (*v*_*A*_, *v*_*B*_), *v*_*A*_ > *v*_*B*_—and the symmetric case (*v*_*A*_, *v*_*B*_), *v*_*B*_ > *v*_*A*_—we fit the curve $C=b\;N^{a}$ and we show the heat-map for the fitted coefficient *a* (bottom-right) and *b* (top-left) across the decision space (see Fig 2 for details). Also in this case we show only configurations where E>0.7. (*A*,*B*) Results for case study I-A with *v*_*i*_ ∈ [0, 1], *γ*_*i*_ = 0.6*v*_*i*_, *α*_*i*_ = 0, *ρ*_*i*_ = 0.1*v*_*i*_, *σ*_*i*_ = 1 and *i* ∈ {A, B} for the homogenous (*A*) and the heterogeneous (*B*) implementation. (*C*,*D*) Results for case study I-B with *v*_*i*_ ∈ [1, 10], *γ*_*i*_ = *ρ*_*i*_ = *v*_*i*_, *α*_*i*_ = 1/*v*_*i*_, *σ*_*i*_ = 10 and *i* ∈ {A, B} for the homogenous (*C*) and the heterogeneous (*D*) implementation.(TIFF)

S3 Fig Mean trajectory of the population fractions Ψ_*A*_, Ψ_*B*_, Ψ_*U*_ over time from the initial condition (Ψ_*A*_ = 0, Ψ_*B*_ = 0, Ψ_*U*_ = 1). Comparison at various levels of abstractions: mean field model (solid lines), macroscopic, finite-size Master equation (circles), and multiagent simulations (triangles). Simulations results are averaged over 100 independent runs. Errorbars are smaller than the symbols size, and are not displayed. (Top) Parameterisation of case study I-A with homogenous multiagent implementation and *v*_*A*_ = 0.9, *v*_*B*_ = 0.6. (Bottom) Parameterisation of case study I-B with heterogenous multiagent implementation and *v*_*A*_ = 9, *v*_*B*_ = 6.
(TIFF)

S4 Fig Micro-macro link with varying number of options for case study I-B. We compare the macroscopic dynamics predicted by the mean-filed model, the finite-size macroscopic dynamics simulated by the Gillespie algorithm and the microscopic dynamics resulting from homogeneous multiagent simulations (*N* = 500 agents). We fix the best option (A) to the maximum quality *v*_*A*_ = 1, and all other options to the same, lower quality *v*_*i*_. The plot shows the fraction of the population committed to option A at the end of the simulation, plotted against the lower option quality *v*_*i*_. Solid lines show the macroscopic prediction of the ODE system. The box-and-whiskers plots represent the statistics from Gillespie and multiagent simulations. Boxes represent the inter-quartile range of the data (2000 runs), while the horizontal lines inside the boxes mark the median values. The whiskers extend to the most extreme data points within 1.5 times the inter-quartile range. Outliers are not shown. A very good match can be appreciated between microscopic and macroscopic dynamics, therefore validating the design pattern for best-of-*n* scenarios.
(TIFF)

S5 Fig Comparison of the macroscopic transition rates resulting from design choices (solid lines) and estimated from the implemented multiagent system (points). The case for distance *d*_*A*_ = *d*_*B*_ = 2.5 m is shown. Estimates have been obtained through survival analysis computing the Nelson-Haelen estimator for the permanence time of agents in each state [29]. Survival analysis provides powerful non-parametric methods to estimate how the probability of events changes over time directly from the experimental data. See also [16] for details.
(TIFF)

S6 Fig Comparison of the macroscopic dynamics with the microscopic homogeneous implementation for all tested decision problems (1000 runs for each setup). We vary both distance *d*_*A*_ ∈ {1.5, 2, 2.5, 3, 3.5} m (from top to bottom) and quality *v*_*a*_ ∈ {0.1, 0.4, 0.7, 1.0} (from left to right), while we keep fixed the distance *d*_*B*_ = 2.5 m and the quality *v*_*B*_ = 1.
(TIFF)

S7 Fig Comparison of the macroscopic dynamics with the microscopic heterogeneous implementation for all tested decision problems (1000 runs for each setup). We vary both distance *d*_*A*_ ∈ {1.5, 2, 2.5, 3, 3.5} m (from top to bottom) and quality *v*_*a*_ ∈ {0.1, 0.4, 0.7, 1.0} (from left to right), while we keep fixed the distance *d*_*B*_ = 2.5 m and the quality *v*_*B*_ = 1.
(TIFF)

S8 Fig Micro-macro link with varying group size for the search and exploitation task. We show results for *v*_*A*_ = 0.7, *v*_*B*_ = 1.0, *d*_*B*_ = 2.5 m and *d*_*A*_ ∈ [1.5 m, 3.5 m]. In this condition, B is the option to be selected, therefore we show the resulting fraction Ψ_*B*_ as the system size *N* varies in [50, 500]. The plot shows results from 2000 simulations for each configuration. Dashed lines represent the equilibrium point of the mean-field model, white boxes represent the master equation approximated through Gillespie simulations, light grey boxes represent homogeneous multiagent simulations, dark grey boxes represent heterogeneous multiagent simulations. Boxes represent the inter-quartile range of the data (2000 runs), while the horizontal lines inside the boxes mark the median values. The whiskers extend to the most extreme data points within 1.5 times the inter-quartile range. Empty circles mark the outliers. A good match between macroscopic and microscopic implementation can be appreciated for every group size. The larger discrepancies are recognised with the heterogeneous implementation for the most difficult decision problem (e.g., the wrong option is also the closest one: *d*_*A*_ = 1.5).
(TIFF)
