# Supplementary material for: A Design Pattern for Decentralised Decision Making
Source: PLoS One. 2015 Oct 23;10(10):e0140950. doi: 10.1371/journal.pone.0140950 (PMC4619747; doi:10.1371/journal.pone.0140950)
Supplement: S1 Text — In this document, we detail and formalise all aspects of the design pattern for decentralised decision making, in some cases repeating and extending the information succinctly given in the main text. (PDF) [file pone.0140950.s001.pdf]

A design pattern for decentralised decision making (S1 Text)

Andreagiovanni Reina[1,◕], Gabriele Valentini[1], Cristian Fernández-Oto[2], Marco Dorigo[1], Vito Trianni[3,◕],

1 IRIDIA, Université Libre de Bruxelles, Brussels, Belgium
2 Faculté des Sciences, Université Libre de Bruxelles, Brussels, Belgium
3 ISTC, Consiglio Nazionale delle Ricerche, Rome, Italy

◕These authors contributed equally to this work.
*** vito.trianni@istc.cnr.it**

General characteristics of design patterns

A design pattern is defined by six attributes: name, problem, context, design rationale, solution, and case-studies [1]. The *name* identifies the design pattern, and should be informative enough to summarise its objective. The *problem* formally describes the purpose of the design pattern, and possibly gives constraints to be satisfied. The *context* determines the domain of applicability of the design pattern, and provides a set of preconditions that must be fulfilled for its usage. The *design rationale* explains the what and the how of the design pattern, that is, its origin and working principles. Typically, the design rationale contains a description of the basic principles underlying the proposed solution, together with some insight about why it efficiently functions. The *solution* provides tools and guidelines for the implementation of the distributed system. The core of the solution is represented by the formal description of the distributed process at different abstraction levels—from macroscopic to microscopic—together with the relationship between them. Additionally, a set of recommended implementation strategies describe how to deal with problems recurring in practical application scenarios, which may have a bearing on the relationship between microscopic and macroscopic description levels (i.e., the micro-macro link). Finally, a design pattern includes a set of *case studies* along with a thorough evaluation to showcase the functioning of the design pattern.

Detailed description of the proposed design pattern

Following the above definition, we provide here a detailed description of the design pattern for decentralised decision making. The case studies are described in the main text. In some cases, the information provided in the main text has been repeated for the sake of completeness.

Name

Collective decisions through cross-inhibition (CDCI)

Problem

The recurring problem tackled by this design pattern is the best-of-n decision problem, that is, the choice of the best option, or any of the equal-best options, among a finite, possibly unknown number n of different alternatives. Each option $i \in \{1, \ldots, n\}$ is characterised by the quality $v_i \in [v_m, v_M]$. The decision-making process therefore requires:

- the identification of (possibly all) the available options;
- the estimation of the quality v_i of each identified option i;
- the selection of the best one, or of any of the equal-best options $i^\star \in \arg\max_{i \in \{1,\ldots,n\}} v_i$.

We study decision making for a distributed system composed of N autonomous agents a_g, $g \in \{1, \ldots, N\}$. Each agent is either committed to one of the available options ($C(a_g) = C_i$, $i \in \{1, \cdots, n\}$), or uncommitted ($C(a_g) = C_U$). At the macroscopic level, a decision is taken as soon as the entire population (or a large fraction Ψ_q) gets committed to one of the options.

The best-of-n decision problem is a challenging problem especially when the number and quality of the available alternatives is not known apriori. Additional complexity might result from uncertain environmental conditions that determine a noisy estimation of the option quality. In other words, the inaccurate quality estimation requires repeated evaluations to increase the decision accuracy. This is a time-consuming process that naturally leads to a speed-accuracy tradeoff [2].

In a distributed system, each agent may discover and evaluate only a small subset of the available options. However, the system as a whole has to converge on the best option (or any of the equal-best options).

Context

The CDCI supports the implementation of decentralised decision-making for a multiagent system in which each agent is autonomous and features the following minimal set of abilities:

- it can individually recognise available options;
- it can individually estimate the options quality;
- it can communicate with peers using small amounts of information;
- it can recognise peers committed to a different option.

Agents neither need to be able to memorise more than one option at a time, nor to explicitly compare different options. The estimated quality $\hat{v}_i$ of a selected option is used only to modulate the individual behaviour (e.g., by altering the probability of performing a certain action). Agents are assumed not to have global knowledge of the system state (e.g., population size, distribution of agents across populations, number of available options). Given these constraints and preconditions, a viable solution to achieve a collective decision is the implementation of a truly decentralised algorithm.

Design rationale

Models of collective decision making have been studied in different domains, from ethology to social dynamics. In this work, we propose a methodology starting from a

model of nest site selection in honeybee swarms [3]. In honeybee swarms, after spring reproduction, several thousands bees leave their hive and create a cluster in the neighbourhood lasting a few days. During this time, the oldest bees in the swarm search for new nest sites and, once they *discover* one, they commit to it. On the one hand, committed bees have a tendency to spontaneously *abandon* their commitment. On the other hand, by interacting with other bees through the waggle-dance, committed bees *recruit* uncommitted nest-mates to the site they have discovered. The waggle-dance duration is proportional to the quality of the advertised nest, and this induces a positive feedback that increases the number of bees committed to the best quality nests. Eventually, a quorum is reached for a single site, which is chosen as the new nest site. Recently, it has been discovered that bees committed to different options *cross-inhibit* each other through stop signals [3]. A bee committed to a site that receives several stop signals abandons its commitment and becomes uncommitted. This mechanism allows the swarm to break decision deadlocks in case of equal-best options. In this way, the swarm reduces the decision time, thus exposure to dangers such as predation or adverse weather conditions.

The decision-making process is based on individual actions and peer-to-peer interactions (i.e., discovery, abandonment, recruitment and cross-inhibition), and lets the swarm quickly converge towards the highest quality option without the need of quality comparisons. It also allows to break deadlocks between same quality options, as well as to modulate the decision dynamics on the basis of the quality of the discovered options [4]. These advantageous characteristics and low requirements in terms of agent capabilities allow designers to apply the design pattern in a large number of different application contexts.

Solution

The collective decision making process of honeybees is modelled as a continuous-time Markov process [3]. Starting from this model, through a mean-field approximation, a deterministic macroscopic model is derived as a system of two coupled ODEs for a binary decision problem. Here, we extend the models for a best-of-n problem and complement the multi-level description by introducing the Master equation, and the Probabilistic Finite State Machine (PFSM) that describes the individual agent behaviour.

Macroscopic description: infinite-size, deterministic, time/state-space continuous Let us consider a population of N agents (with $N \to \infty$). At the macroscopic level, we model the population fractions of committed agents $\Psi_i = N_i/N$ (with N_i the number of agents committed to option i) and the fraction of uncommitted agents $\Psi_U = N_U/N$ (with N_U the number of uncommitted agents). Agents change their commitment state through four different processes: discovery (γ), abandonment (α), recruitment (ρ) and cross-inhibition (σ).

We extend the model for binary decisions proposed in [3] to the best-of-n decision problem. The model describes the mean system behaviour as a system of n coupled ODEs and an algebraic equation for mass conservation:

$$\begin{cases} \dot{\Psi}_i &= \gamma_i \Psi_U - \alpha_i \Psi_i + \rho_i \Psi_i \Psi_U - \sum_{j \neq i} \sigma_j \Psi_i \Psi_j \\ \Psi_U &= 1 - \sum_i \Psi_i \end{cases} , \quad i \in \{1, \ldots, n\} \tag{1}$$

Each differential equation in (1) describes the variation of the fraction of agents in each population. The fraction of agents committed to option i increases through discovery (at a rate γ_i) and through recruitment proportional to the population committed to i (at a rate $\rho_i \Psi_i$). Conversely, the fraction decreases through abandonment (at a rate α_i)

or through cross-inhibition proportional to the contrasting populations (at a rate $\sum_{j\neq i}\sigma_j\Psi_j,$). All model parameters represent the rate at which agents change their commitment state. Therefore, we assume all model parameters to be non-negative:

$$\alpha_i, \gamma_i, \rho_i, \sigma_i \geq 0 \tag{2}$$

For a decision-making problem based on the quality of the available options, all model parameters could be linked to the option quality v_i:

$$\alpha_i = f_\alpha(v_i),\ \gamma_i = f_\gamma(v_i),\ \rho_i = f_\rho(v_i),\ \sigma_i = f_\sigma(v_i), \tag{3}$$

where each function describes a specific relationship between transition rate and option quality (see [4] for an example).

This model describes the average proportion of agents in each population for a system with an infinite number of agents. It is deterministic and continuous in time and in the state space. The model can be exploited to determine the macroscopic behaviour corresponding to a given parameterisation, and to provide constraints to the possible parameterisations in order to obtain a desired system behaviour. This ultimately translates in constraints in the design of the relationship between option quality v_i and transition rates γ_i, α_i, ρ_i, and σ_i.

Macroscopic description: finite-size, stochastic, time continuous, state-space discrete The mean-field model can be derived from a Markov process describing the dynamics of a population with finite size N [3]. We can represent the generalised case for best-of-n decisions through chemical reactions representing agents changing their commitment state, either spontaneously or by interacting with other agents:

$$\begin{aligned} C_U &\xrightarrow{\mathcal{Q}_{\gamma_i}} C_i \\ C_i &\xrightarrow{\mathcal{Q}_{\alpha_i}} C_U \\ C_U + C_i &\xrightarrow{\mathcal{Q}_{\rho_i}} 2C_i \\ C_i + C_j &\xrightarrow{\mathcal{Q}_{\sigma_j}} C_U + C_j, \qquad i \neq j, \end{aligned} \tag{4}$$

where the $\mathcal{Q}_{\lambda_i}, \lambda \in \{\alpha, \gamma, \rho, \sigma\}$ represent reaction constants [5]. Starting from the above description, it is possible to derive the master equation, which describes the time evolution of the system as a stochastic, discrete-state process. More precisely, the master equation describes the time evolution of the probability mass function related to each possible state in which the system can be found:

$$\frac{\delta}{\delta t} P(\mathbf{N}, t) = \sum_{k=1}^{4n} [\beta_k - P(\mathbf{N}, t)\mathcal{Q}_k], \quad \forall \mathbf{N} \tag{5}$$

where $\mathbf{N} = \langle N_U, N_1, \ldots, N_n \rangle$ corresponds to the system state, k is an index for each of the $4n$ possible transitions, and the term β_k is the probability that the system is one transition k "away" from state $\mathbf{N}$ at time t, and undergoes the transition k in $(t, t + \delta t)$. The quantities $\mathcal{Q}_k$ are defined as follows:

$$\begin{array}{rlcrl} \mathcal{Q}_1 = & N_U \mathcal{Q}_{\gamma_1} & & \mathcal{Q}_2 = & N_1 \mathcal{Q}_{\alpha_1} \\ \mathcal{Q}_3 = & N_U N_1 \mathcal{Q}_{\rho_1} & & \mathcal{Q}_4 = & \sum_{j\neq 1} N_1 N_j \mathcal{Q}_{\sigma_j} \\ & . & . & & . \\ & . & . & & . \\ & . & . & & . \\ \mathcal{Q}_{4n-3} = & N_U \mathcal{Q}_{\gamma_n} & & \mathcal{Q}_{4n-2} = & N_n \mathcal{Q}_{\alpha_n} \\ \mathcal{Q}_{4n-1} = & N_U N_n \mathcal{Q}_{\rho_n} & & \mathcal{Q}_{4n} = & \sum_{j\neq n} N_n N_j \mathcal{Q}_{\sigma_n} \end{array} \tag{6}$$

For instance, in the binary case with options A and B, the term $N_U N_A \mathcal{Q}_{\rho_A} \delta t$ represents the probability that a recruitment transition for option A occurs in the time interval δt, changing the system state from $\langle N_U, N_A, N_B \rangle$ to $\langle N_U - 1, N_A + 1, N_B \rangle$.

The transition rates of the ODE model of equation (1) have direct correspondence with the transition probabilities of the master equation (5). For the generic transition rate λ, the conversion formula is:

$$\mathcal{Q}_{\lambda_i} = \lambda_i N^{1-n_a}, \qquad \begin{array}{r} \lambda \in \{\gamma, \alpha, \rho, \sigma\} \\ i \in \{1, \cdots, n\} \end{array} \tag{7}$$

where n_a is the number of populations involved in the transition. The factor N^{1-n_a} is consequence of the fact that the transition rates are used in differential equations that contain population fractions, while the transition probabilities are used in combination with the total number of agents in the population. For transitions involving a single population, i.e., discovery and abandonment, we have $n_a = 1$ and therefore we obtain a direct correspondence between transition rates and probabilities per unit time:

$$\mathcal{Q}_{\gamma_i} = \gamma_i, \qquad \mathcal{Q}_{\alpha_i} = \alpha_i \tag{8}$$

Conversely, for recruitment and cross-inhibition (i.e., transitions that correspond to interactions between populations), $n_a = 2$ and therefore

$$\mathcal{Q}_{\rho_i} = \rho_i N^{-1}, \qquad \mathcal{Q}_{\sigma_i} = \sigma_i N^{-1} \tag{9}$$

These relations provide a mean to link the two macroscopic descriptions of the process, allowing to study the adherence of the finite-size system to the mean-field dynamics.

At this description level, the model accounts for the stochastic fluctuations of the system due to finite-size effects (i.e., the influence of a finite system size N). Given the complexity of analytically solving the master equation (5), we analyse it through numerical simulation via the Gillespie algorithm [5]. As we show in the main text, the numerical analysis reveals how the system behaviour departs from the predictions of the mean-field approximation. This model allows us to study the effects of the relationship between quality v and transition probabilities $\mathcal{Q}_\lambda$, and therefore to take decisions about the desired macroscopic dynamics at design time.

Microscopic description: agent-based, stochastic, time/state-space discrete
The average agent behaviour is modelled as a probabilistic finite state machine (PFSM, see panel A in S1 Fig.) with $n+1$ states $\{C_U, C_1, \ldots, C_n\}$ which represent the agent commitment state, and by $4n$ transition probabilities (four transitions for each option i), which determine the state change (either spontaneous or upon interaction with agents of a different population). Differently from the previous models, here the system changes state at discrete time steps of length τ. The $4n$ probabilities determine the individual behaviour and may be modulated according to the option quality v_i.

Upon discovery of option i, agents make a transition from state C_U to state C_i with probability $\mathcal{P}_\gamma(v_i)$ (in average). Similarly, upon abandonment of option i, agents make a transition from state C_i to state C_U with probability $\mathcal{P}_\alpha(v_i)$. Discovery and abandonment are spontaneous transitions, that is, the probability depends solely on the option quality v_i (as estimated by the agent itself). Conversely, the remaining transitions depend also on the size of the different sub-populations, which can be estimated upon interaction with other agents (see below). Recruitment to option i is modelled by a transition from state C_U to state C_i with probability $\mathcal{P}_{\Psi_i} \mathcal{P}_\rho(v_i)$, where the first factor accounts for the probability of interacting with agents already committed to option i given the current population size, and the second factor accounts for a quality-dependent probability of triggering the state change. Similarly, cross-inhibition

is modelled by a transition from state C_i to state C_U with probability $\sum_{j\neq i} \mathcal{P}_{\Psi_j}\mathcal{P}_\sigma(v_j)$. Here, the overall transition probability aggregates the probability of interaction with any agent committed to option $j \neq i$. Also in this case, the first factor accounts for the population size and the second factor accounts for a quality-dependent transition probability. The cross-inhibition of an agent committed to option i is influenced by the population size and by the quality of the contrasting options.

As already mentioned, at this level we describe the agent behaviour in average, which might not correspond to the actual implementation. In fact, the transition probabilities between commitment states might be implemented differently for each agent, as we will show in the following sections. The transition probabilities $\mathcal{P}_\lambda(v_i), \lambda \in \{\gamma, \alpha, \rho, \sigma\}$ presented above correspond to the average case, and they can be related to the probabilities per unit time of executing a transition at the macroscopic level, so that the following relations hold:

$$\begin{array}{lll} \mathcal{P}_\gamma(v_i) & = \mathcal{Q}_{\gamma_i}\tau & = \gamma_i\tau \\ \mathcal{P}_\alpha(v_i) & = \mathcal{Q}_{\alpha_i}\tau & = \alpha_i\tau \\ \mathcal{P}_{\Psi_i}\mathcal{P}_\rho(v_i) & = \mathcal{Q}_{\rho_i}N_i\tau & = \rho_i\tau N_i N^{-1} \\ \mathcal{P}_{\Psi_i}\mathcal{P}_\sigma(v_i) & = \mathcal{Q}_{\sigma_i}N_i\tau & = \sigma_i\tau N_i N^{-1} \end{array} \tag{10}$$

where τ is the discrete time step of the PFSM. Under the assumption of a well-mixed system, the probability of interaction with an agent committed to option i corresponds to the fraction Ψ_i, that is:

$$\mathcal{P}_{\Psi_i} = \frac{N_i}{N} \tag{11}$$

Considering also Eq. (3), we can derive a general quality-dependent relationship for the transition probability:

$$\lambda_i = f_\lambda(v_i) \ \rightarrow \ \mathcal{P}_\lambda(v_i) = f_\lambda(v_i)\tau, \qquad \begin{array}{c} \lambda \in \{\gamma, \alpha, \rho, \sigma\} \\ i \in \{1, \ldots, n\} \end{array} \tag{12}$$

To obtain a desired macroscopic behaviour, one can opportunely define the average transition probabilities as a function of the quality v_i. Conversely, given the relationship between quality and individual probabilities, it is possible to easily derive the macroscopic dynamics.

Implementation guidelines To proceed to the implementation of the agent behaviour, several *design choices* are required to determine how agents change state depending on either the population size or the option quality. The challenge is given by the fact that agents do not have access to global information—e.g., population size, number of available options—as described in the CDCI design pattern context. In such conditions, it is necessary to make design choices about the strategy for executing the state transitions of the PFSM to guarantee a one-to-one correspondence between the microscopic and macroscopic description levels.

Population size dependent probabilities The computation of the probability $\mathcal{P}_{\Psi_i}$ requires a decentralised estimation of the population size given that, in the considered distributed system, neither N_i or N are available to the individual agent. Each agent compensates this lack of knowledge by estimating a probability P_{Ψ_i} through interactions with neighbours. A possible solution consists in letting each agent take a sample of the total population. This means that before taking action, the agent has to collect enough information about the size of the different populations by sampling the state of neighbour agents:

$$P_{\Psi_i} = \frac{|\tilde{\mathcal{A}}_i|}{|\tilde{\mathcal{A}}|}, \tag{13}$$

where $\tilde{\mathcal{A}}$ is the set of sampled agents, and $\tilde{\mathcal{A}}_i \subseteq \tilde{\mathcal{A}}$ is the set of agents in state C_i. Depending on the pattern of interactions and on the sample size $|\tilde{\mathcal{A}}|$, the quality of the estimation largely varies. Therefore, it is necessary to carefully design the sampling size in order to guarantee a certain level of accuracy.

Another possibility is to let each agent draw a random agent $a_{\hat{g}}$ from its local neighbourhood, check its commitment state and compute the probability P_{Ψ_i} as:

$$P_{\Psi_i} = \begin{cases} 1 & C(a_{\hat{g}}) \in C_i \\ 0 & C(a_{\hat{g}}) \notin C_i \end{cases}, \qquad i \in \{1, \cdots, n\} \tag{14}$$

Assuming a well-mixed system, the frequency of picking an agent committed to option i is given exactly by Eq. (11), which corresponds to the desired behaviour in average. This second strategy is more parsimonious, as it requires fewer agent-agent interactions and no additional computations, and is therefore the choice that maximises speed.

Homogeneous versus heterogeneous implementation For what concerns the other transition probabilities ($\mathcal{P}_\gamma$, $\mathcal{P}_\alpha$, $\mathcal{P}_\rho$, $\mathcal{P}_\sigma$), we propose two strategies based on either homogeneous or heterogeneous system implementation. In the homogeneous case, all agents share the same transition probability, leading to a direct correspondence between the actual and the average agent behaviour:

$$P_{\lambda,g}(v_i) = \mathcal{P}_\lambda(v_i), \qquad \begin{array}{c} g \in \{1, \dots, N\} \\ \lambda \in \{\gamma, \alpha, \rho, \sigma\} \\ i \in \{1, \dots, n\} \end{array} \tag{15}$$

From (12), we can derive a first constraint to respect for a correct system implementation, following from the need that each probability must be less than 1:

$$P_{\lambda,g}(v_i) \leq 1 \;\rightarrow\; f_\lambda(v_i) \leq \frac{1}{\tau} \tag{16}$$

In the heterogeneous case, each agent a_g computes independently its own transition probability $P_{\lambda,g}(v_i)$, and the system behaviour results from the aggregation of the individual responses. At the macroscopic level, the transition probability per unit time (or conversely the transition rate) depends on the probability that any agent in a given population follows the corresponding transition. This depends on both the way in which the individual agent follows a transition, and on the heterogeneity of the system. In order to relate the macroscopic parameters to the individual probabilities and the option quality v_i, we propose to implement the transition probabilities with a simple response threshold scheme. The agent a_g follows a transition with a fixed probability if the (estimated) option quality exceeds a given response threshold δ_g:

$$P_{\lambda,g}(v_i) = \begin{cases} \mathcal{P}_{\lambda\uparrow} & v_i > \delta_g \\ \mathcal{P}_{\lambda\downarrow} & v_i \leq \delta_g \end{cases}, \tag{17}$$

where $\mathcal{P}_{\lambda\uparrow}$ and $\mathcal{P}_{\lambda\downarrow}$ are tuneable parameters, and the value δ_g is drawn for each agent a_g from a probability distribution $\mathcal{D}_\lambda$ over the range $[v_m, v_M]$. With this implementation, it is possible to establish a relationship between microscopic and macroscopic parameters through the cumulative distribution function of $\mathcal{D}_\lambda$, $F_{\mathcal{D}_\lambda}$:

$$F_{\mathcal{D}_\lambda} = \frac{\mathcal{P}_\lambda - \mathcal{P}_{\lambda\downarrow}}{\mathcal{P}_{\lambda\uparrow} - \mathcal{P}_{\lambda\downarrow}} \tag{18}$$

For $F_{\mathcal{D}_\lambda}$ to be a cumulative distribution function, it is required that the relationship between quality and macroscopic transition rate expressed in Eq. (3) be monotonic in

v—either increasing or decreasing. As a consequence, the step function (17) can be determined by:

$$\begin{array}{l} \mathcal{P}_{\lambda\uparrow} = \mathcal{P}_\lambda(v_M) = f_\lambda(v_M)\tau \\ \mathcal{P}_{\lambda\downarrow} = \mathcal{P}_\lambda(v_m) = f_\lambda(v_m)\tau \end{array}, \tag{19}$$

which together with Eq. (12) provides the micro-macro link for the heterogeneous case.

Such a micro-macro link holds when each agent a_g re-samples the threshold δ_g from $\mathcal{D}_\lambda$ at every decision step. From an implementation perspective, however, re-sampling is not a parsimonious design choice, neither it is biologically plausible. Instead, fixed thresholds would be a more suitable solution: they would simplify the design and are also biologically relevant (e.g., response thresholds determined genetically or acquired through learning [6]). However, fixed thresholds lead to a quasi-deterministic behaviour of the agents in face of a given option quality, as they are unable to modulate their behaviour according to the perceievd quality. The decision problem would therefore lead to "frozen" sub-populations, and the microscopic dynamics would diverge from the macroscopic predictions. By studying the behaviour of different parameterisation, we recognised that an approximation with fixed thresholds is still valid for recruitment and cross-inhibition, because re-sampling is ensured by changing partner in each different interaction (as shown in case study I-A). Instead, the micro-macro link is hampered by the usage of fixed thresholds for spontaneous transitions, unless the macroscopic dynamics are dominated by recruitment and cross-inhibition (as shown in case study 1-B). Therefore, a principled choice about the usage of fixed thresholds can be made on the basis of the desired macroscopic patarmeterisation. Should the system be governed principally by spontaneous transitions, the fixed threshold scheme is not suitable. Otherwise, it represents a viable solution, which is also biologically plausible [7]. Finally, note that homogeneous and heterogeneous strategies can be mixed together, so that the agent behaviour can be homogeneous with respect to some transition probabilities, and heterogeneous with respect to others.

Latent and interactive agents As a further implementation guideline, we discuss here the case in which agents cannot interact every τ seconds. This is a very common condition in practical application scenarios, because of spatial/topological factors that determine the interaction pattern, or because interactions are constrainted by limitations of the computing power or by the communication channel. To model such conditions, we introduce the possibility for agents to be either latent or interactive. When an agent is latent, it cannot communicate or receive messages from neighbours, but is still capable of changing its commitment state following spontaneous transitions. In the interactive state, agents are capable of communicating with other agents, and therefore can change commitment state accordingly. We refer to changes in the latent/interactive state as *activity dynamics*, as opposed to the commitment dynamics resulting into changes of the commitment state. We model the activity dynamics by considering that an agent becomes latent with probability $\mathcal{P}_L$ and returns interactive with probability $\mathcal{P}_I$ (see also the PFSM in Fig. 1B in the main text). Equivalently, agents may remain in the interactive or latent state for exponentially distributed time intervals, respectively with mean time $\tau_I = 1/\mathcal{P}_L$ and $\tau_L = 1/\mathcal{P}_I$. Under these conditions, the distribution of agents between interactive and latent states reaches asymptotically the fractions $\eta_I = \mathcal{P}_I/(\mathcal{P}_I + \mathcal{P}_L)$ and $\eta_L = \mathcal{P}_L/(\mathcal{P}_I + \mathcal{P}_L)$.

By coupling together activity and commitment dynamics, we obtain a microscopic description with $2(n+1)$ states. Here, agents can be uncommitted and latent (state C_U^L), uncommitted and interactive (state C_U^I), committed to option $i \in \{1, \ldots, n\}$ and latent (state C_i^L), or committed to i and interactive (state C_i^I). Transitions between these states can be arranged in different ways, constrained by the need to correctly represent both the commitment and the activity dynamics. Recruitment and

cross-inhibition are available only when agents are interactive, and the final state can be either interactive or latent, depending on the application. Conversely, discovery and abandonment may be available in any state, and the actual choice depends on the application needs. An example PFSM is provided in panel B of S1 Fig., and corresponds to the microscopic description for the search and exploitation task described in this paper. Here, the transition probabilities between different activity states must be appropriately tuned to reduce to the overall activity dynamics described by the PFSM of Fig. 1B in the main text. In the given example, the following relations hold:

$$\begin{array}{c} \mathcal{P}_{L,i} = \mathcal{P}_L - \sum_{j \neq i} \mathcal{P}_{\Psi_j} \mathcal{P}_{\sigma_j} \\ \mathcal{P}_{L,U} = \mathcal{P}_L - \sum_i \mathcal{P}_{\Psi_i} \mathcal{P}_{\rho_i} \end{array} , \qquad i \in \{1, \ldots, n\}, \tag{20}$$

which ensure that the overall transition probability from interactive to latent states sums up to $\mathcal{P}_L$. Note that in the PFSM of S1 Fig. panel B, there is always just one possible transition from a latent to an interactive state, with probability $\mathcal{P}_I$.

In order to maintain a micro-macro link despite the existence of latent agents, the population of interactive agents must always be an unbiased sample of the entire population. More precisely, given the fraction Ψ^I (Ψ^L) of agents in the interactive state I (latent state L), we require that:

$$\frac{\Psi_i^I}{\Psi^I} \approx \frac{\Psi_i^L}{\Psi^L} \approx \Psi_i, \tag{21}$$

where Ψ_i^I and Ψ_i^L represent the fractions of agents that are found in state C_i^I and C_i^L within the entire population. In fact, if changes in the commitment state within the interactive sub-population (fraction Ψ^I) are much faster than changes in the activity state (i.e., agents switching between states I and L), the distribution of commitment states among interactive agents would misrepresent the global population distribution, and therefore the microscopic and macroscopic dynamics would diverge. As a consequence, we require that the transitions in the activity state must be faster than transitions in the commitment state, for instance by constraining the decision to change commitment state to each transition from latent to interactive, as done in the search and exploitation task presented in this paper.

The correspondence between microscopic and macroscopic parameters depends on the way in which microscopic transitions are implemented. Because some transitions are available only to interactive (latent) agents, the corresponding macroscopic rate must be reduced by η_I (η_L), which represent the fraction of the population that can actually change commitment state. Conversely, given a desired macroscopic transition rate, the average probabilities per agent must be increased by $1/\eta_I$ ($1/\eta_L$). For the example of S1 Fig. panel B, recruitment and cross-inhibition transitions are available only when agents are interactive, therefore Eq. (12) should be written as follows:

$$\lambda_i = f_\lambda(v_i) \;\rightarrow\; \mathcal{P}_\lambda(v_i) = \frac{f_\lambda(v_i)\tau}{\eta_I}, \qquad \begin{array}{c} \lambda \in \{\rho, \sigma\} \\ i \in \{1, \ldots, n\} \end{array} \tag{22}$$

On the other hand, discovery and abandonment are only available to latent agents, and therefore the average probability per agent must take into account the proportion of agents in the latent state:

$$\lambda_i = f_\lambda(v_i) \;\rightarrow\; \mathcal{P}_\lambda(v_i) = \frac{f_\lambda(v_i)\tau}{\eta_L}, \qquad \begin{array}{c} \lambda \in \{\gamma, \alpha\} \\ i \in \{1, \ldots, n\} \end{array} \tag{23}$$

Minimum speed of the process The time step τ at which the agent updates its commitment state determines the process speed. In order to obtain a precise

correspondence between macroscopic transition rates and microscopic transition probabilities, the value of τ must be conveniently sized. To calculate an upper bound for τ, we consider the coexistence of transitions exiting from a same state of the PFSM, and we require that the total probability of leaving the state be lower than one. For instance, an agent that directly follows the behaviour described by the PFSM of S1 Fig. panel A has n recruitment and n discovery transitions all exiting from state C_U. This means that the overall probability from all outgoing transitions must not exceed one:

$$\sum_{i=1}^{n} \mathcal{P}_\gamma(v_i) + \mathcal{P}_{\Psi_i}\mathcal{P}_\rho(v_i) \leq 1 \tag{24}$$

Similarly, a constraint is given by the overall outgoing probability from the commitment state C_i:

$$\mathcal{P}_\alpha(v_i) + \sum_{j\neq i}^{n} \mathcal{P}_{\Psi_j}\mathcal{P}_\sigma(v_j) \leq 1 \tag{25}$$

Recall however that the PFSM of S1 Fig. panel A is the representation of the average agent, which differs from the actual implementation. The implementation guidelines described above prescribe that at most one interactive transition is available at a time. Additionally, we can assume that at most one spontaneous transition may become available at a time, given that in most application scenarios the evaluation of available alternatives is performed sequentially by individual agents. Overall, to compute the upper bound, we consider only one interactive and one spontaneous transitions at a time. A safe upper bound of τ is guaranteed by considering the extreme case in which $\mathcal{P}_{\Psi_i} = 1$ and v_i maximizes $\mathcal{P}_\lambda(v_i)$ (with $\lambda \in \{\gamma, \alpha, \rho, \sigma\}$):

$$\begin{cases} \max_{v_i} \mathcal{P}_\rho(v_i) + \max_{v_i} \mathcal{P}_\gamma(v_i) \leq 1 \\ \max_{v_i} \mathcal{P}_\alpha(v_i) + \max_{v_i} \mathcal{P}_\sigma(v_i) \leq 1 \end{cases}, \tag{26}$$

which, together with (12), can be rewritten as follows:

$$\begin{cases} \tau \leq \left(\max_{v_i} f_\rho(v_i) + \max_{v_i} f_\gamma(v_i)\right)^{-1} \\ \tau \leq \left(\max_{v_i} f_\alpha(v_i) + \max_{v_i} f_\sigma(v_i)\right)^{-1} \end{cases} \tag{27}$$

The constraint for the upper limit of the agent's time-step τ reduces to the minimum value of Eq. (27). Note that in Eq. (27), we specify a constraint on the multi-agent system speed as a function of only macroscopic transition rates independently of the implementation strategy of the individual agent behaviour (whether homogenous, heterogenous or mixed). This upper-bound can be further refined considering the actual implementation, and the possible existence of latent and interactive states.

Dealing with episodic discovery Discovery is the process that allows to report the existence of an option to the swarm. In many practical application scenarios, discovery is an episodic event, that is, a given option is recognised by the agents only occasionally due to the limited individual agent capabilities. This can be related either to temporal or spatial constraints (e.g., discovery of options is correlated with the position in space of the agent, see the search and exploitation task presented in the main text). When no option is available, the agent cannot make a discovery transition. This has a bearing on the macroscopic dynamics resulting from the agent behaviour, because the macroscopic rate depends on the probability that an agent actually encounters the option i. If we refer to this probability as E_i, then we can rewrite Eq. (12) as follows:

$$\gamma_i = f_\gamma(v_i) \;\rightarrow\; \mathcal{P}_\gamma(v_i) = \frac{f_\gamma(v_i)\tau}{E_i} \tag{28}$$

Knowing how agents discover potential options is necessary to correctly link the microscopic and the macroscopic dynamics. In many practical scenarios, E_i can be estimated a priori to support the choice of the microscopic parameterisation.

References

1. Babaoğlu G, Canright G, Deutsch A, Di Caro GA, Ducatelle F, Gambardella LM, et al. Design Patterns from Biology for Distributed Computing. ACM Transactions on Autonomous and Adaptive Systems. 2006;1(1):26–66.

2. Chittka L, Skorupski P, Raine NE. Speed-accuracy tradeoffs in animal decision making. Trends Ecol Evol. 2009;24(7):400–407.

3. Seeley TD, Visscher PK, Schlegel T, Hogan PM, Franks NR, Marshall JAR. Stop signals provide cross inhibition in collective decision-making by honeybee swarms. Science. 2012;335(6064):108–11.

4. Pais D, Hogan PM, Schlegel T, Franks NR, Leonard NE, Marshall JAR. A Mechanism for Value-Sensitive Decision-Making. PLoS ONE. 2013;8(9):e73216.

5. Gillespie DT. Exact stochastic simulation of coupled chemical reactions. J Phys Chem A. 1977;81(25):2340–2361.

6. Jeanson R, Weidenmüller A. Interindividual variability in social insects - proximate causes and ultimate consequences. Biol Rev Camb Philos Soc. 2013 Dec;89(3):671–687.

7. Robinson EJH, Franks NR, Ellis S, Okuda S, Marshall JAR. A Simple Threshold Rule Is Sufficient to Explain Sophisticated Collective Decision-Making. PLoS ONE. 2011;6(5):e19981.
