# Supplementary material for: A Design Pattern for Decentralised Decision Making
Source: PLoS One. 2015 Oct 23;10(10):e0140950. doi: 10.1371/journal.pone.0140950 (PMC4619747; doi:10.1371/journal.pone.0140950)
Supplement: S2 Text — In this document, we provide additional results supporting case study I-A, in particular concerning the stability analysis of the macroscopic system and the selection of the desired macroscopic parameterisation. (PDF) [file pone.0140950.s002.pdf]

A design pattern for decentralised decision making (S2 Text)

Andreagiovanni Reina[1,◕], Gabriele Valentini[1], Cristian Fernández-Oto[2], Marco Dorigo[1], Vito Trianni[3,◕],

1 IRIDIA, Université Libre de Bruxelles, Brussels, Belgium
2 Faculté des Sciences, Université Libre de Bruxelles, Brussels, Belgium
3 ISTC, Consiglio Nazionale delle Ricerche, Rome, Italy

◕These authors contributed equally to this work.
*** vito.trianni@istc.cnr.it**

Stability analysis and parameterisation choice for multiagent simulations on fully-connected networks

To determine the parameters of case study I-A, we analyse the macroscopic ODE model. To meet the requirement of consensus decision, the model must display equilibria for the points $S_A = \{\Psi_A = 1, \Psi_B = 0\}$ and $S_B = \{\Psi_A = 0, \Psi_B = 1\}$. The system has equilibria S_A and S_B for $\alpha_A = \alpha_B = 0$. We choose $\sigma_A = \sigma_B = 1$, and we perform a stability analysis of the system as a function of the parameters γ_i, ρ_i, $i \in \{A, B\}$. Given the domain space:

$$\begin{cases} 0 \leq \Psi_i \leq 1, & i \in \{A, B\} \\ 0 \leq \Psi_U \leq 1 \\ \Psi_A + \Psi_B + \Psi_U = 1 \end{cases}, \tag{1}$$

the analysis of the macroscopic ODE model reveals three possible equilibria: S_A, S_B and S_X. Equilibria S_A and S_B do not change position and are always present for any parameterisation but change stability. In contrast, equilibrium S_X is always unstable and appears only when S_A and S_B are both stable, as a function of the parameters γ_i and ρ_i. The stability analysis gives:

$$\begin{array}{lcl} S_A \text{ is stable} & \Leftrightarrow & \gamma_B < \gamma_A + \rho_A \\ S_B \text{ is stable} & \Leftrightarrow & \gamma_A < \gamma_B + \rho_B \end{array} \tag{2}$$

The equilibria S_A and S_B change stability through transcritical bifurcations, and when one of the consensus solutions is unstable, the other one is the unique stable solution.

Given Eq. (2), we can parametrise the system to minimise the chance of wrong decisions. We require that there exists only one stable solution for a quality difference above a target resolution R:

$$|v_A - v_B| / \max(v_A, v_B) > R \tag{3}$$

Let us assume $v_A > v_B$. On the one hand S_A must be the only stable solution, and therefore S_B should be unstable, hence $\gamma_A > \gamma_B + \rho_B$. On the other hand, the constraint on the target resolution implies that $v_A - v_B/v_A > R$. We select linear functions that link macroscopic transition rates to quality:

$$\gamma_i = f_\gamma(v_i) = k\, v_i \quad \rho_i = f_\rho(v_i) = h\, v_i, \quad i \in \{\mathrm{A}, \mathrm{B}\} \tag{4}$$

where k and h are tuneable parameters. To satisfy our design choices, we combine the above equations and solve the system:

$$\begin{cases} k\,v_A & > k\,v_B + h\,v_B \\ v_A - v_B > v_A R \end{cases} \quad \rightarrow \quad k > h\,(1-R)/R \tag{5}$$

An identical relation can be obtained assuming $v_B > v_A$. We arbitrarily select the target resolution $R = 0.15$, and we finally select a parameterisation that complies with the prescribed bounds: $h = 0.1$ and $k = 0.6$.
