# Supplementary figures and images for: A Design Pattern for Decentralised Decision Making
Source: PLoS One. 2015 Oct 23;10(10):e0140950. doi: 10.1371/journal.pone.0140950 (PMC4619747; doi:10.1371/journal.pone.0140950)

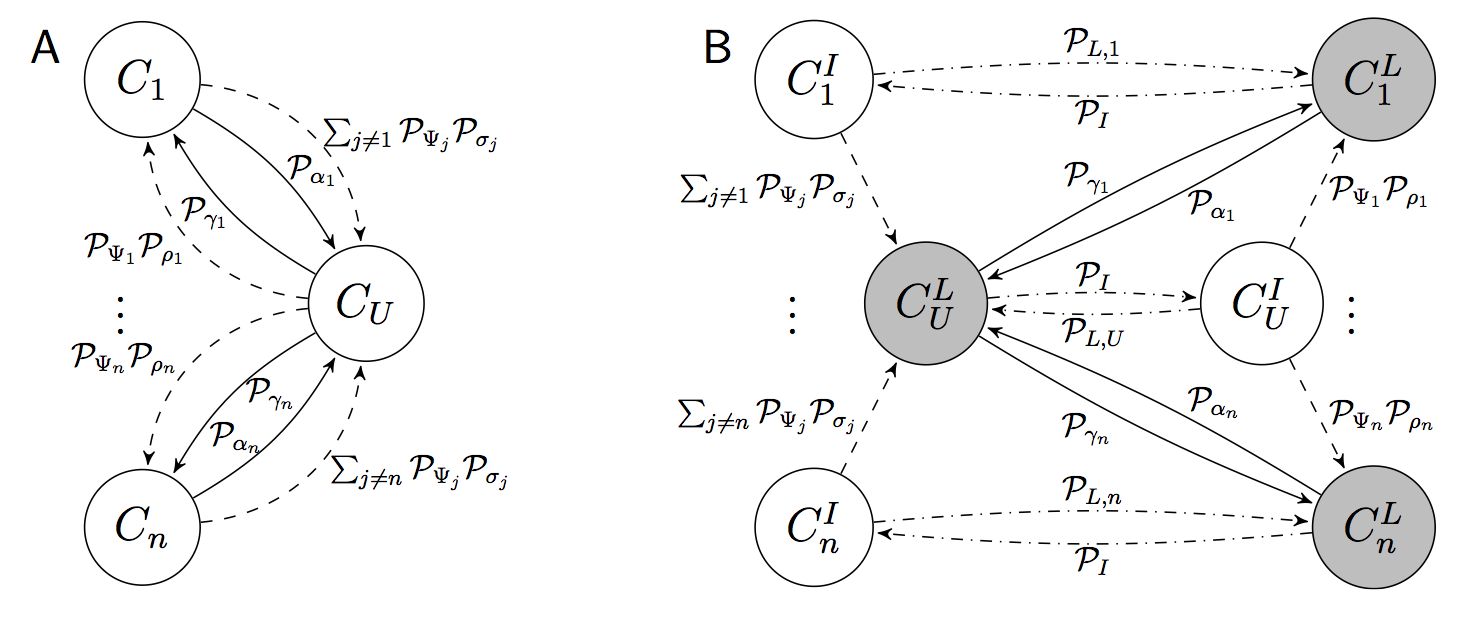

Supplement: S1 Fig — Here, the notation Pλi,λ∈{γ,α,ρ,σ}, i ∈ {1, …, n} is a shorthand for Pλ(vi). (A) PFSM describing the basic commitment dynamics for n possible options. Spontaneous transitions are represented by solid lines, while interactive transitions are represented by dashed lines. (B) PFSM describing the coupled commitment and activity dynamics. Latent states are indicated in grey, and dash-dotted lines represent changes between latent and interactive states. (TIFF) [file pone.0140950.s003.tiff]

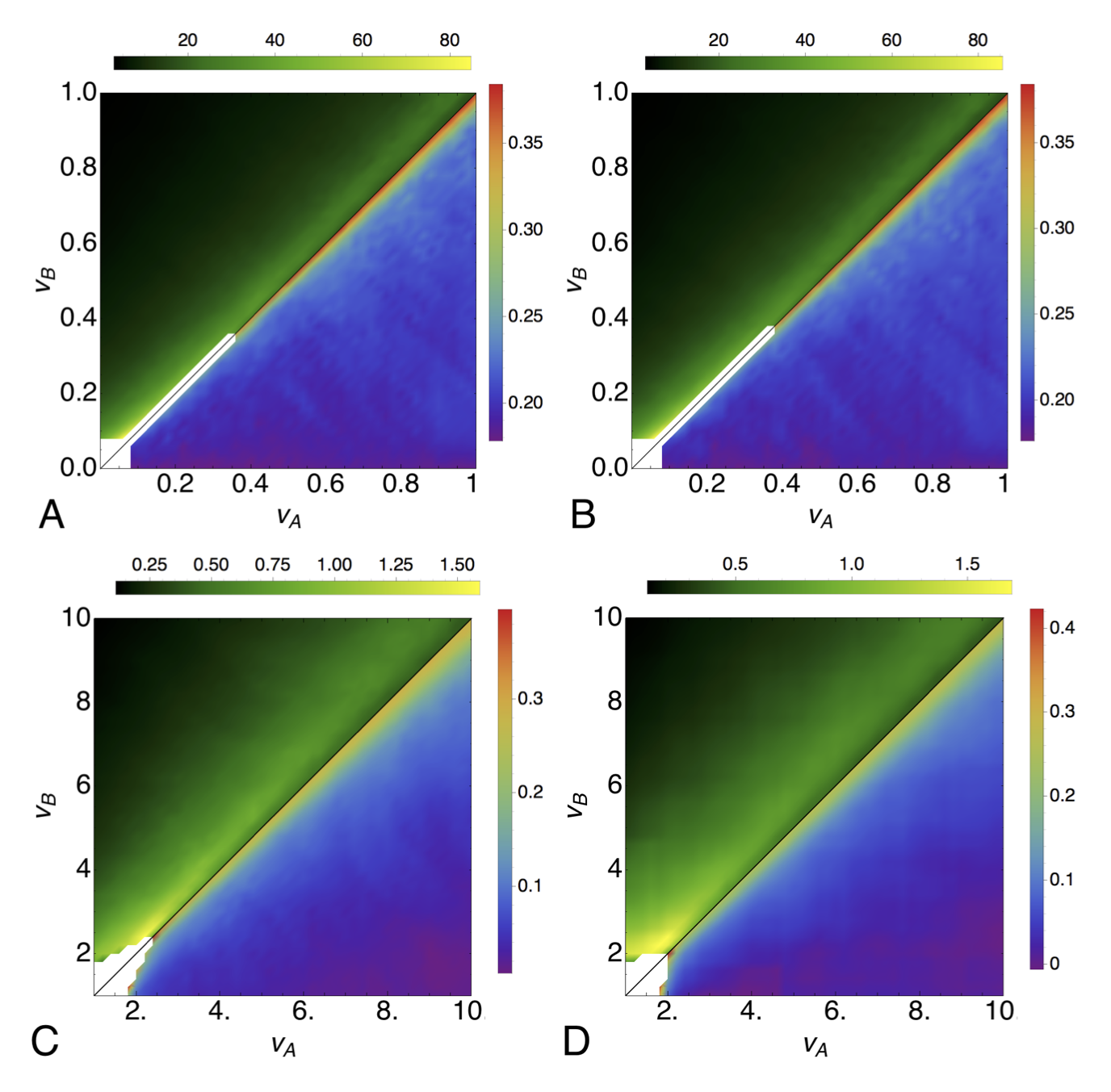

Supplement: S2 Fig — For each configuration (v A, v B), v A > v B—and the symmetric case (v A, v B), v B > v A—we fit the curve C=bNa and we show the heat-map for the fitted coefficient a (bottom-right) and b (top-left) across the decision space (see Fig 2 for details). Also in this case we show only configurations where E>0.7. (A,B) Results for case study I-A with v i ∈ [0, 1], γ i = 0.6v i, α i = 0, ρ i = 0.1v i, σ i = 1 and i ∈ {A, B} for the homogenous (A) and the heterogeneous (B) implementation. (C,D) Results for case study I-B with v i ∈ [1, 10], γ i = ρ i = v i, α i = 1/v i, σ i = 10 and i ∈ {A, B} for the homogenous (C) and the heterogeneous (D) implementation. (TIFF) [file pone.0140950.s004.tiff]

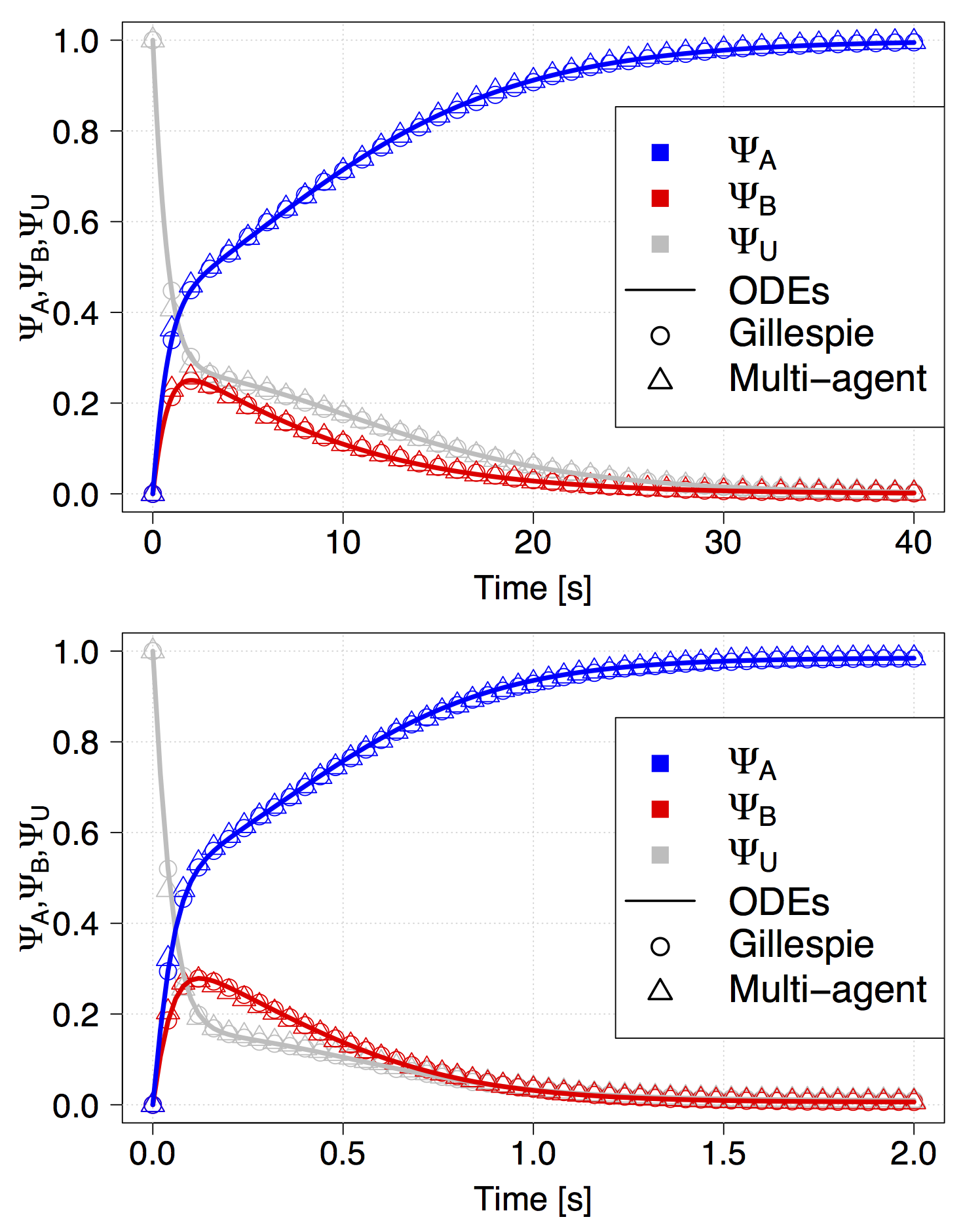

Supplement: S3 Fig — Comparison at various levels of abstractions: mean field model (solid lines), macroscopic, finite-size Master equation (circles), and multiagent simulations (triangles). Simulations results are averaged over 100 independent runs. Errorbars are smaller than the symbols size, and are not displayed. (Top) Parameterisation of case study I-A with homogenous multiagent implementation and v A = 0.9, v B = 0.6. (Bottom) Parameterisation of case study I-B with heterogenous multiagent implementation and v A = 9, v B = 6. (TIFF) [file pone.0140950.s005.tiff]

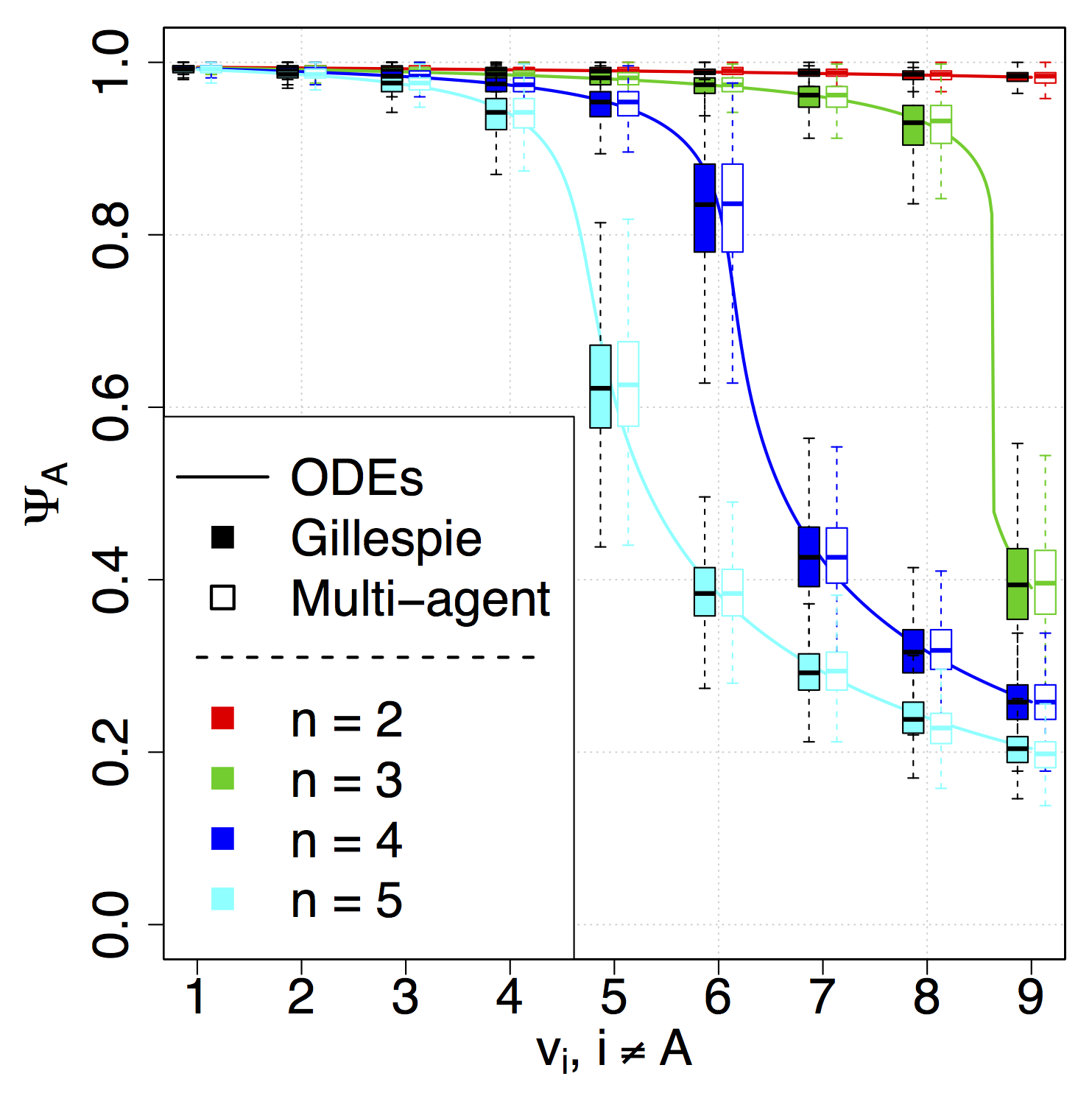

Supplement: S4 Fig — We compare the macroscopic dynamics predicted by the mean-filed model, the finite-size macroscopic dynamics simulated by the Gillespie algorithm and the microscopic dynamics resulting from homogeneous multiagent simulations (N = 500 agents). We fix the best option (A) to the maximum quality v A = 1, and all other options to the same, lower quality v i. The plot shows the fraction of the population committed to option A at the end of the simulation, plotted against the lower option quality v i. Solid lines show the macroscopic prediction of the ODE system. The box-and-whiskers plots represent the statistics from Gillespie and multiagent simulations. Boxes represent the inter-quartile range of the data (2000 runs), while the horizontal lines inside the boxes mark the median values. The whiskers extend to the most extreme data points within 1.5 times the inter-quartile range. Outliers are not shown. A very good match can be appreciated between microscopic and macroscopic dynamics, therefore validating the design pattern for best-of-n scenarios. (TIFF) [file pone.0140950.s006.tiff]

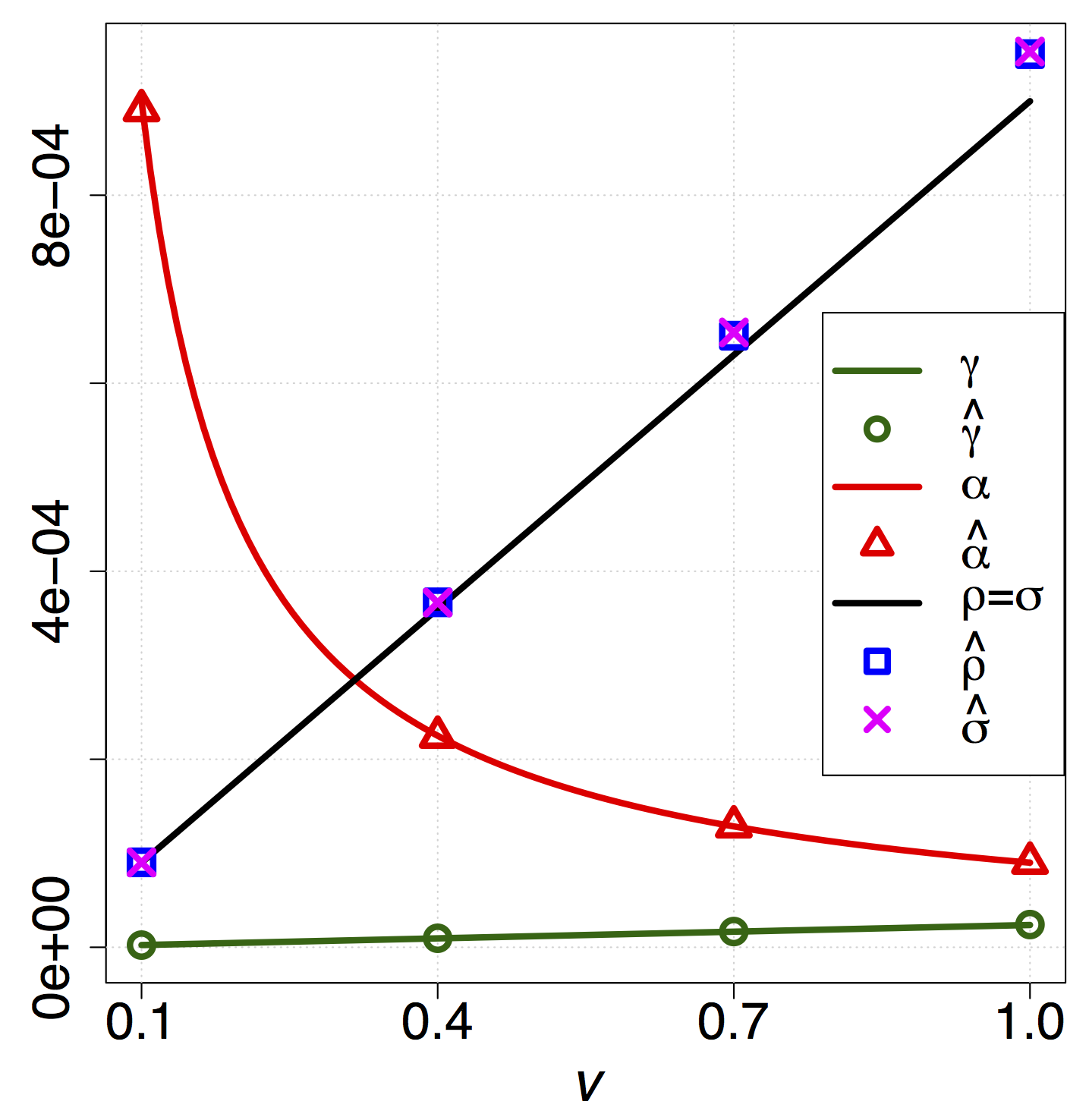

Supplement: S5 Fig — The case for distance d A = d B = 2.5 m is shown. Estimates have been obtained through survival analysis computing the Nelson-Haelen estimator for the permanence time of agents in each state [29]. Survival analysis provides powerful non-parametric methods to estimate how the probability of events changes over time directly from the experimental data. See also [16] for details. (TIFF) [file pone.0140950.s007.tiff]

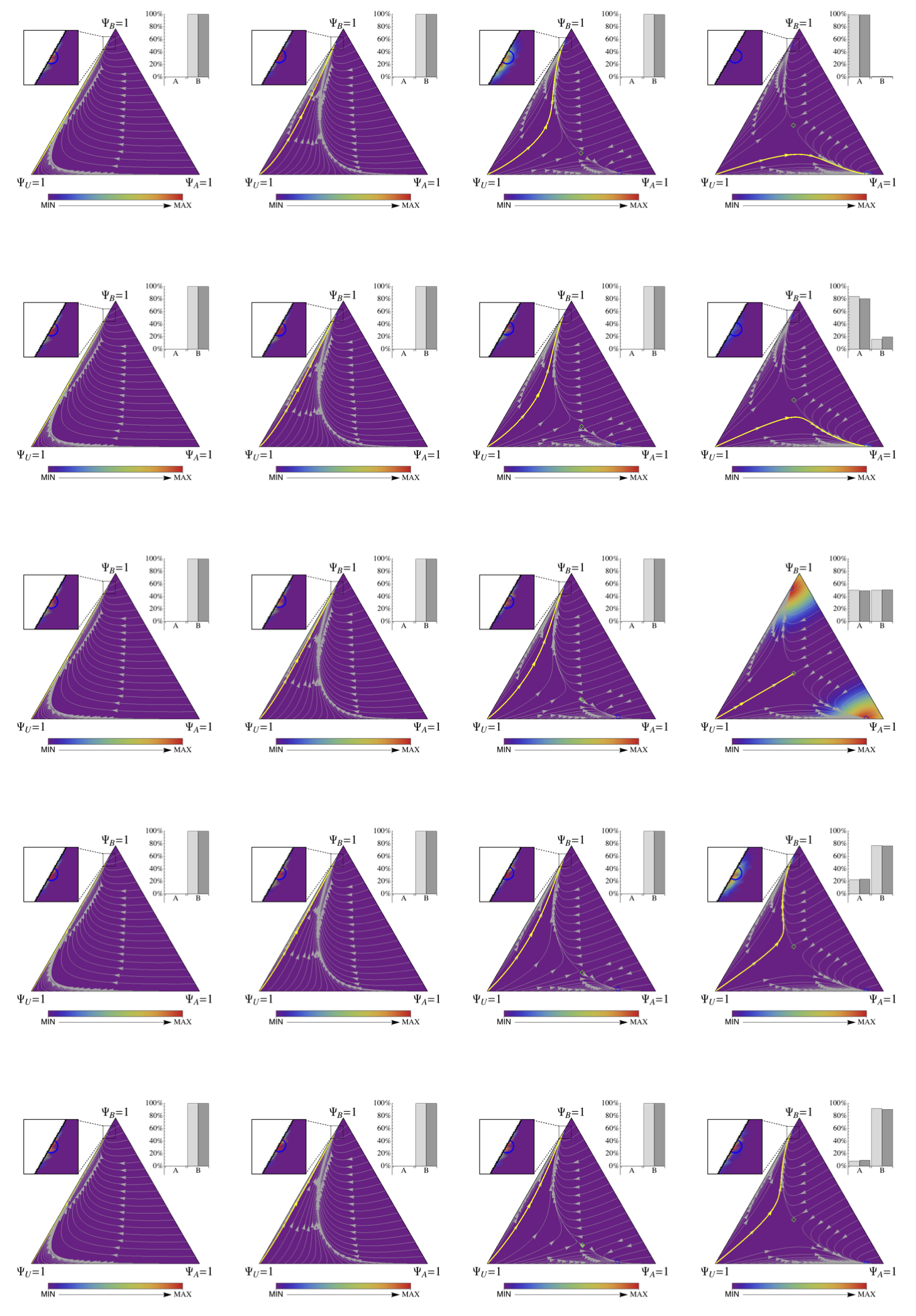

Supplement: S6 Fig — We vary both distance d A ∈ {1.5, 2, 2.5, 3, 3.5} m (from top to bottom) and quality v a ∈ {0.1, 0.4, 0.7, 1.0} (from left to right), while we keep fixed the distance d B = 2.5 m and the quality v B = 1. (TIFF) [file pone.0140950.s008.tiff]

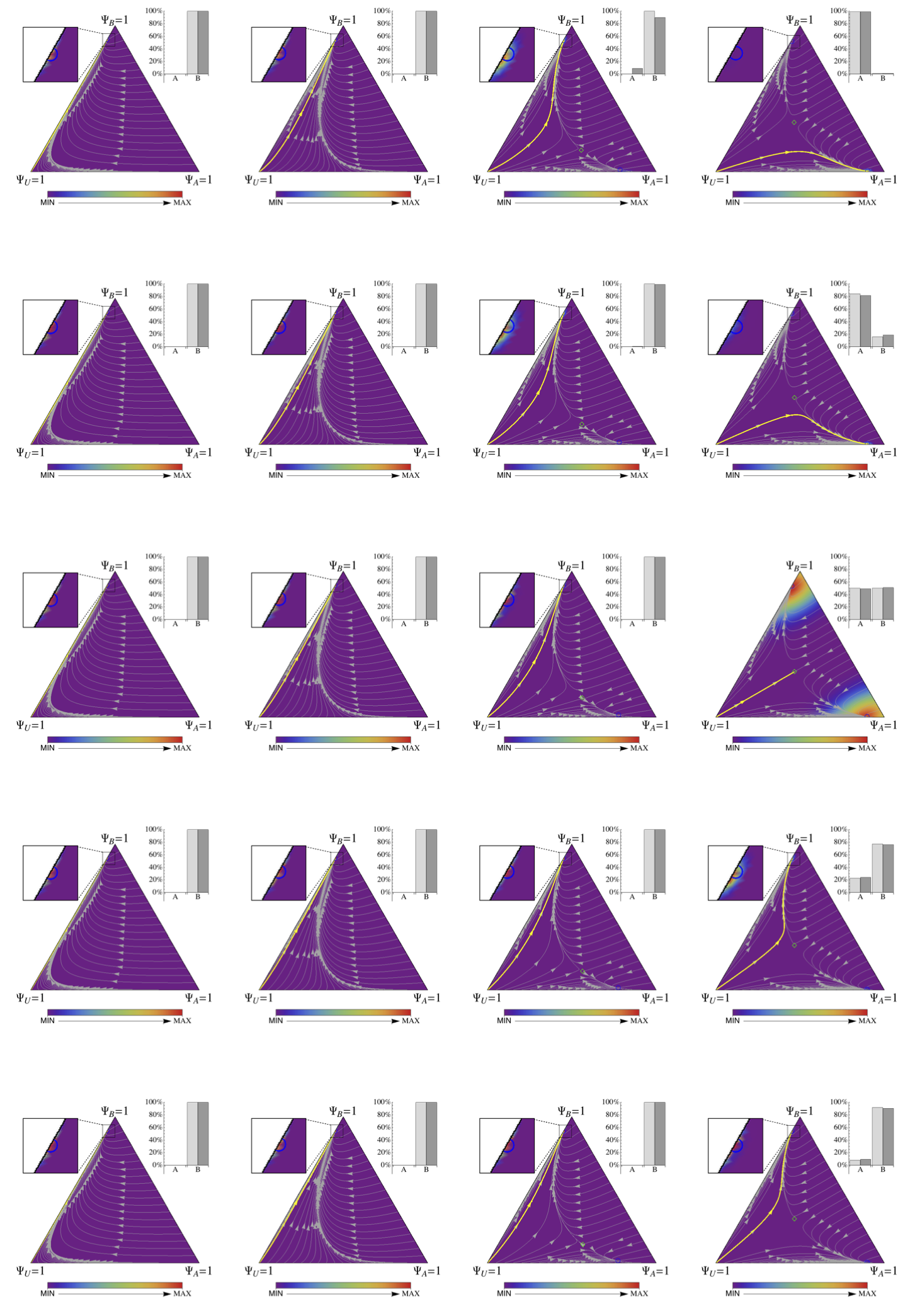

Supplement: S7 Fig — We vary both distance d A ∈ {1.5, 2, 2.5, 3, 3.5} m (from top to bottom) and quality v a ∈ {0.1, 0.4, 0.7, 1.0} (from left to right), while we keep fixed the distance d B = 2.5 m and the quality v B = 1. (TIFF) [file pone.0140950.s009.tiff]

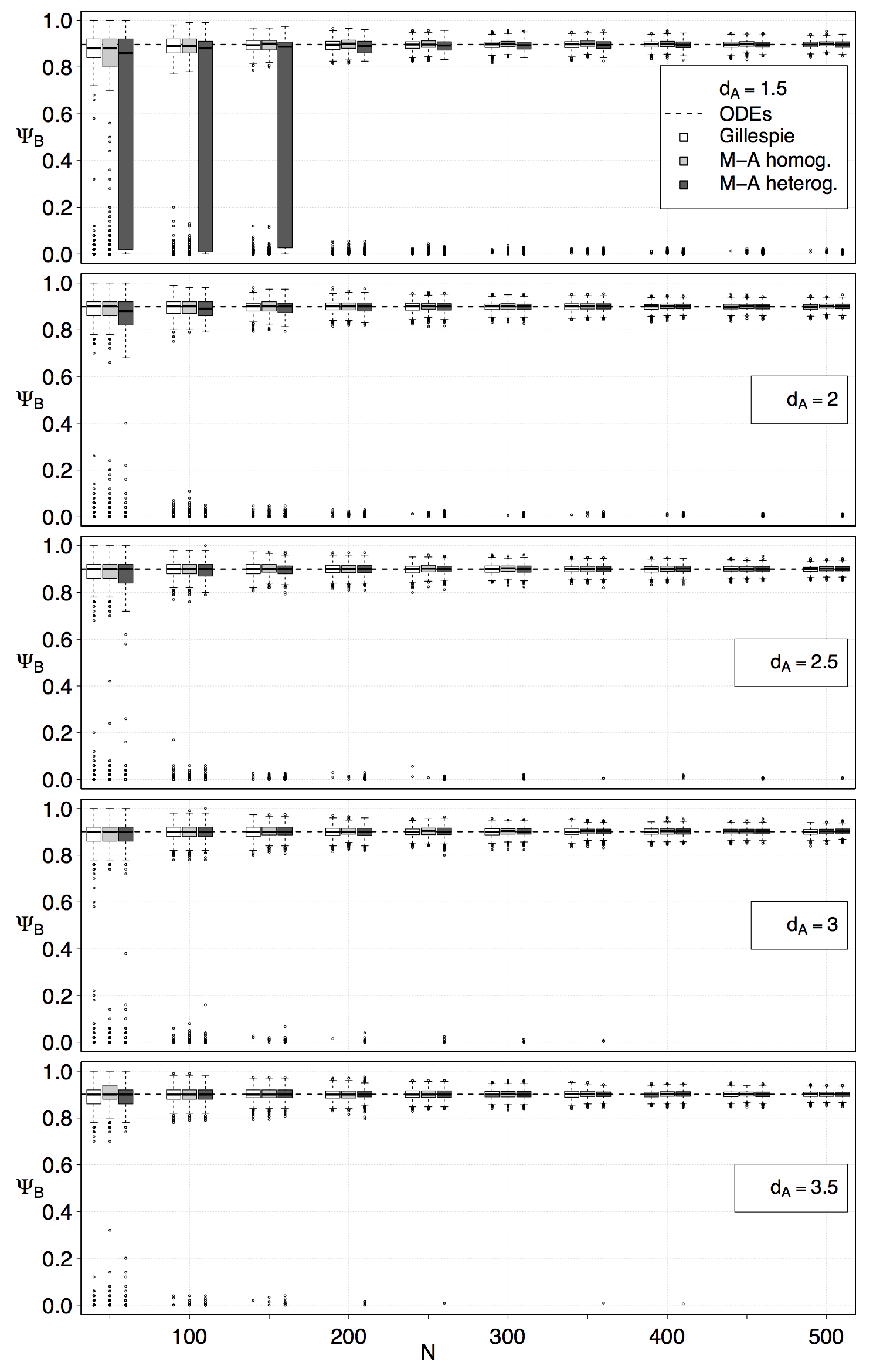

Supplement: S8 Fig — We show results for v A = 0.7, v B = 1.0, d B = 2.5 m and d A ∈ [1.5 m, 3.5 m]. In this condition, B is the option to be selected, therefore we show the resulting fraction ΨB as the system size N varies in [50, 500]. The plot shows results from 2000 simulations for each configuration. Dashed lines represent the equilibrium point of the mean-field model, white boxes represent the master equation approximated through Gillespie simulations, light grey boxes represent homogeneous multiagent simulations, dark grey boxes represent heterogeneous multiagent simulations. Boxes represent the inter-quartile range of the data (2000 runs), while the horizontal lines inside the boxes mark the median values. The whiskers extend to the most extreme data points within 1.5 times the inter-quartile range. Empty circles mark the outliers. A good match between macroscopic and microscopic implementation can be appreciated for every group size. The larger discrepancies are recognised with the heterogeneous implementation for the most difficult decision problem (e.g., the wrong option is also the closest one: d A = 1.5). (TIFF) [file pone.0140950.s010.tiff]
